# Deconstruction of Neurotrypsin Reveals a Multi-factorially Regulated Activity Affecting Myotube Formation and Neuronal Excitability

**DOI:** 10.1007/s12035-022-03056-2

**Published:** 2022-10-05

**Authors:** Anselmo Canciani, Cristina Capitanio, Serena Stanga, Silvia Faravelli, Luigi Scietti, Lisa Mapelli, Teresa Soda, Egidio D’Angelo, Pascal Kienlen-Campard, Federico Forneris

**Affiliations:** 1 The Armenise-Harvard Laboratory of Structural Biology, Department of Biology and Biotechnology, University of Pavia, Via Ferrata 9/A, 27100 Pavia, Italy; 2 Present Address: Molecular Machines and Signaling, Max Planck Institute of Biochemistry, 82152 Martinsried, Germany; 3 Aging and Dementia Research Group, CEMO Department, Institute of Neuroscience, UCLouvain, B-1200 Brussels, Belgium; 4 Present Address: Neuroscience Institute Cavalieri Ottolenghi, 10043 Orbassano, TO Italy; 5 Present Address: Department of Neuroscience Rita Levi Montalcini, University of Turin, 10126 Turin, Italy; 6 Present Address: Biochemistry and Structural Biology Unit, Department of Experimental Oncology, IRCCS European Institute of Oncology (IEO), Via Adamello 16, 20139 Milan, Italy; 7 Department of Brain and Behavioral Sciences, University of Pavia, Via Forlanini 6, 27100 Pavia, Italy; 8 IRCCS Mondino Foundation, Via Mondino 2, Pavia, Italy

**Keywords:** Synapses, Neurotrypsin, Neuromuscular junction, Serine protease, Agrin signaling, Proteolytic regulation

## Abstract

Neurotrypsin (NT) is a highly specific nervous system multi-domain serine protease best known for its selective processing of the potent synaptic organizer agrin. Its enzymatic activity is thought to influence processes of synaptic plasticity, with its deregulation causing accelerated neuromuscular junction (NMJ) degeneration or contributing to forms of mental retardation. These biological effects are likely to stem from NT-based regulation of agrin signaling. However, dissecting the exact biological implications of NT-agrin interplay is difficult, due to the scarce molecular detail regarding NT activity and NT-agrin interactions. We developed a strategy to reliably produce and purify a catalytically competent engineered variant of NT called “NT-mini” and a library of C-terminal agrin fragments, with which we performed a thorough biochemical and biophysical characterization of NT enzyme functionality. We studied the regulatory effects of calcium ions and heparin, identified NT’s heparin-binding domain, and discovered how zinc ions induce modulation of enzymatic activity. Additionally, we investigated myotube differentiation and hippocampal neuron excitability, evidencing a dose-dependent increase in neuronal activity alongside a negative impact on myoblast fusion when using the active NT enzyme. Collectively, our results provide in vitro and cellular foundations to unravel the molecular underpinnings and biological significance of NT-agrin interactions.

**Supplementary Information:**

The online version contains supplementary material available at 10.1007/s12035-022-03056-2.

## Introduction

Cell-to-cell communication interfaces, like chemical synapses, are responsible for the vital processes of signal transmission mediating both neuromuscular communication and higher brain functions. The correct organization of processes underlying their formation and maintenance is critical and relies on coordinated signaling pathways integrating an array of mechanical, molecular, electrical, and chemical signals [1–3]. In this context, proteolytic processing of synaptic organizers by specific proteases can serve as a potent tool to spatio-temporally modulate those pathways. This can also function as a mechanism to ensure synchronized communication and organization of pre- and postsynaptic architectures [2, 4]. Several proteases have been described as contributing to such regulatory mechanisms, with differing degrees of specificity [5]. Among these, neurotrypsin [6] (NT, also known as PRSS12, motopsin [7], and leydin [8]) stands out for its specificity [9] and its possible biological roles [10–12] that would make it a modulator of both central and peripheral nervous system (CNS, PNS) synaptic plasticity.

Discovered in the late 1990s, this nervous system multi-domain cysteine-rich serine protease was initially described on the basis of predictive sequence analyses [6, 7, 13]. Produced predominantly by neurons of the CNS and PNS, NT expression peaks in the perinatal period, a timeframe often associated with intense synaptogenesis and synaptic rearrangement [14]. In adult organisms, NT expression is maintained at a lower level and remains the highest within the CNS [1, 14]. Interestingly, NT is known to accumulate in synaptic vesicles and to be released in the extracellular environment in an activity-dependent manner [15]. When secreted in the synapse, NT cleaves with high specificity its only known substrate, the large proteoglycan agrin, at two distinct sites (named α and β) proximate to its C-terminus, yielding two fragments of 22 and 90 kDa. This calcium-dependent process is modulated by glycosaminoglycans (such as heparin [16, 17]), seems to play an important role in synaptic rearrangement influencing CNS synaptic plasticity [10, 15], and contributes to the destabilization of the nerve-muscle contacts at neuromuscular junctions (NMJs) [11, 18]. Accordingly, coordination of pre- and postsynaptic signaling seems to regulate NT activity in the CNS, hinting at a role in processes of activity-driven synaptic plasticity and long-term potentiation essential for learning and memory [4, 10, 19]. Deregulation of NT was seen to affect CNS plasticity and contribute to NMJ-related neurodegenerative disorders. Namely, inactivation leads to a form of non-syndromic mental retardation [12], while overactivity induces accelerated aging and destabilization of NMJs leading to sarcopenia (a muscle wasting disease of the elderly) [11, 20, 21]. Interestingly, NT might even play a role in other major CNS neurodegenerative disorders, such as Alzheimer’s disease (AD), as its expression and activity were tentatively linked to presenilins [22], which are key players in AD onset and progression.

**Fig. 1:**
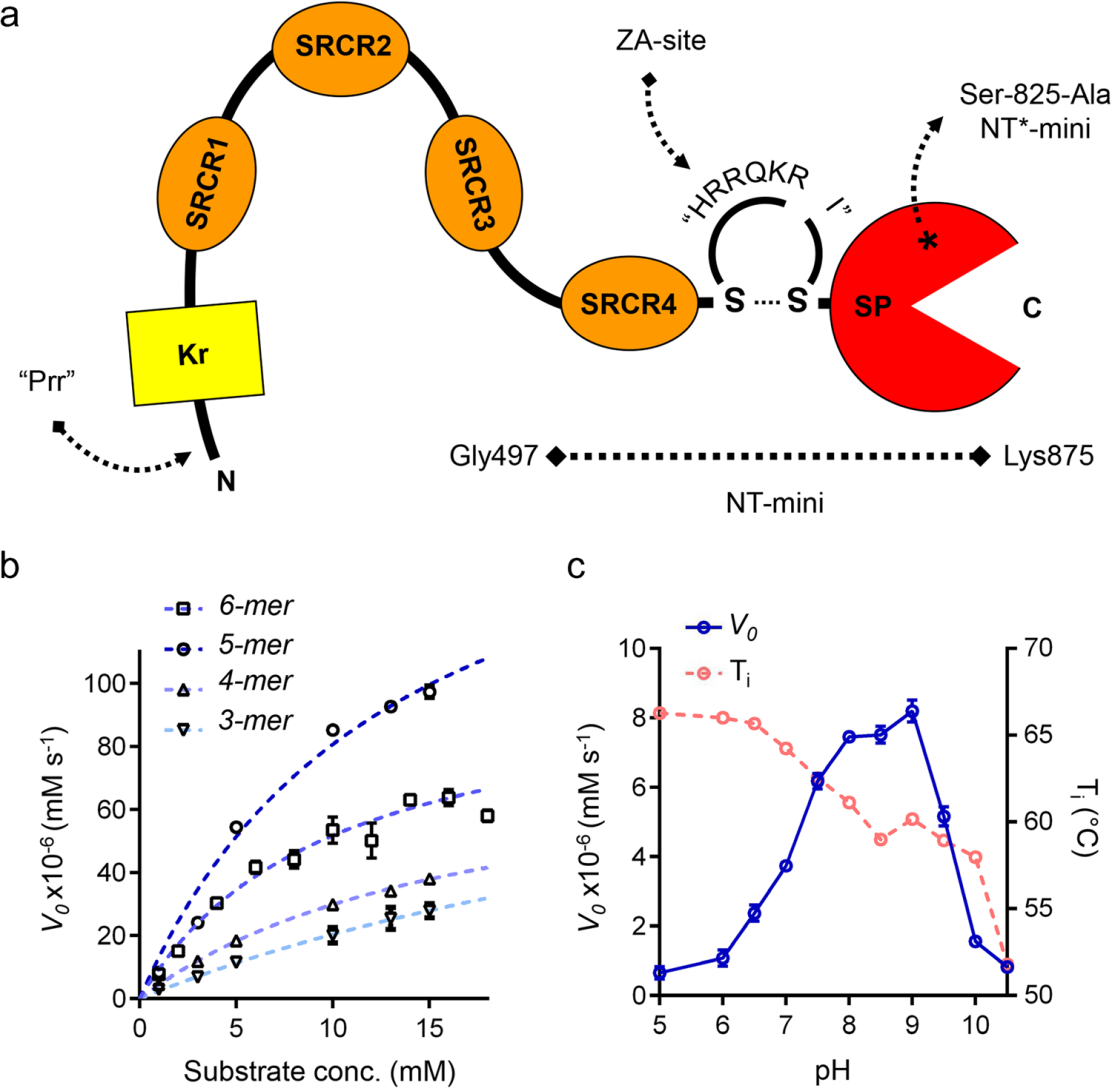
Representation of NT-mini and characterization of its enzymatic activity using synthetic peptides. **a** Schematic representation of neurotrypsin (NT) showing boundaries of NT-mini (Gly497-Lys875). Prr, proline-rich region; Kr, kringle domain; SRCR 1–4, scavenger receptor cysteine-rich domains 1–4; SP, serine protease domain; ZA, zymogen activation site. Asterisk indicates the inactivating Ser825Ala mutation introduced to generate NT*-mini. **b** Michaelis–Menten plot of para-nitroaniline (pNa) conjugated β-peptide (KGLVEK) substrates of decreasing lengths (6-mer to 3-mer). *K*_m_ and *k*_cat_ were estimated using non-linear regression (dashed lines) Michaelis–Menten enzyme-kinetics models in Prism 6.01. Error bars represent the standard deviation (SD) of averaged data points from triplicate independent experiments (*n* = 3). **c** Variation of NT-mini activity and stability in function of increasing pH. Changes in *V*_0_ processing at a fixed 1 mM 6-mer β-peptide substrate displayed in blue (solid line); alterations in Nano-DFS determined thermal stability (*T*_i_) shown in red (dashed line). Error bars represent the standard deviation (SD) of averaged data points from triplicate independent experiments (*n* = 3)

NT’s domain composition is inferred from the original sequence analyses, which highlighted the presence of a proline-rich N-terminal region followed by a kringle (Kr) domain, 3–4 scavenger receptor cysteine-rich (SRCR1-4) domains, a predicted Furin-recognized zymogen activation (ZA) site, and a C-terminal catalytic serine protease (SP) domain [6, 13] (Fig. 1a). Experimental data mostly describes the catalytic processing of agrin by NT, providing a broad outline of its properties while highlighting the unusually different makeup of its α (-GPPVER/A-) and β (-KGLVEK/S-) substrate cleavage sites. While data regarding NT’s catalytic activity has allowed the description of several features of its SP domain, the function of the remaining domains is still a matter of debate. Of its 4–5 accessory domains (Kr/SRCR1-4), only the Kr and murine SRCR3 domains have been characterized and associated with putative functions [23, 24]. The former was associated with the binding of a seizure-related protein of the CNS (sez-6) [25], while the latter was found to directly mediate binding to calcium [24], essential to NT’s activity [16].

Despite the relative amount of information supporting NT’s possible biological roles, specific knowledge regarding its molecular architecture/function is scarce. This constitutes an intrinsic limitation to our understanding of NT functionality, something which is particularly relevant considering how its interplay with agrin might underpin NT’s role in physiological and pathological processes. In particular, a strong limitation to our understanding of NT functionality lies in the challenging recombinant production of this enzyme, which requires complex approaches (murine hybridomas) and yields modest sample amounts [16] hardly compatible with the requirements of extensive biochemical characterizations.

To address the knowledge gaps surrounding the NT-agrin axis, we developed a novel strategy to recombinantly produce a catalytically competent fragment of human NT (named NT-mini), as well as its catalytically inactive Ser825Ala mutant (NT*-mini), potentially capable of recapitulating the functions of the full-length NT “parent.” To better investigate NT catalytic processing, we generated a library of recombinant C-terminal human agrin fragments including the various splicing insertions critical for NMJ signaling [26, 27]. The biochemical, biophysical, and cellular characterization of these molecules highlighted how NT-mini can recapitulate the functions of full-length NT without the need for its N-terminal accessory domains. Moreover, we found that NT-mini is capable of enhancing the excitability of hippocampal neurons, hinting that the full protein could have a role in the activity-dependent modulation of neuronal networks. Collectively, our work both offers new insights into NT-agrin interplay and provides a novel framework to address the details of this complex system and its pathophysiological implications.

## Materials and Methods

### Molecular Cloning

Recombinant constructs for NT-mini (Gly497-Leu875) and AGR-46 (Pro1635-Pro2068) agrin-like substrates were amplified using a Phusion DNA polymerase (Thermo Fisher Scientific) starting from a source synthetic gene (GeneWiz) corresponding the full-length human NT cDNA (UniProt P56730-1) and from a human agrin y0z0 cDNA (UniProt O00468-6) obtained from Source Biosciences, respectively, using the oligonucleotides listed in Sup. Table 1.

The resulting PCR products were subcloned into pCR4-TOPO vectors (Thermo Fisher Scientific) using BamHI and NotI restriction sites. These plasmids served as the basis for the generation of the inactivated Ser-825-Ala NT*-mini and of the various AGR-46 agrin splice variant combinations via site-directed mutagenesis. The corresponding PCR reactions were performed with Phusion DNA polymerase (Thermo Fisher Scientific) using the oligonucleotides listed in Sup. Table 1.

The longer agrin AGR-124 construct was designed as a codon-optimized cDNA synthetic gene (GeneWiz) for mammalian expression, spanning human agrin y4z19 (Uniprot O00468-1) FS9-LG3 domains (Ala893-Pro2068).

All constructs were verified using Sanger sequencing (Microsynth) and then were subcloned into modified pUPE.106.08 plasmids (U-Protein Express BV) for secreted protein expression in mammalian cell culture systems. For NT-mini, the final expression constructs contained an N-terminal 8xHis-SUMO tag used to stimulate expression and facilitate purification. For agrin constructs, the final expression constructs contained an N-terminal 6xHis-tag followed by a tobacco etch virus (TEV) protease cleavage site.

### Recombinant Protein Production

All NT and agrin constructs were produced via transient transfection following the protocols described in [28], but using SFM-HEK293 (Expression Systems) cell suspension cultures adapted to grow in a serum-free medium (ESF, Expression Systems). Transfections were performed using linear polyethylenimine (PEI MAX, Polysciences) in a 5:1 (w:w) PEI:DNA ratio. Zymogen-to-enzyme activation of NT-mini constructs was performed during protein production by co-transfecting the NT-mini plasmid in a 1:5 ratio with a pUPE expression plasmid for mammalian expression (U-Protein Express BV) carrying the human cDNA of the pro-protein convertase Furin. Four hours post-transfection, the culture was supplemented with peptone supplement (Primatone RL, Sigma-Aldrich) at a final concentration of 0.6% (*w*/*v*). Transfected cultures were maintained for 6 days to allow recombinant protein expression and accumulation. On the 7th day, the growth media was harvested by centrifugation. The cells were discarded and the recombinant proteins were purified from the supernatant using liquid chromatography.

### Recombinant Protein Purification

Cell media were adjusted with concentrated buffer to a final 200 mM NaCl, 25 mM HEPES/NaOH, and pH 8 and filtered through a 0.45-µm syringe filter (Sarstedt) using a peristaltic pump (Thermo Fisher Scientific). All chromatographic steps were carried out using an NGC chromatography system (Biorad), and followed by monitoring UV absorbance at 280 nm. Samples from each step were further analyzed with reducing and non-reducing SDS-PAGE. Purification of NT-mini or NT*-mini was obtained with a three-step Heparin/Ni-IMAC “mixed-mode” approach and completed by size exclusion chromatography (SEC). The filtered medium was supplemented with 5 mM CaCl_2_ (final concentration) and loaded, at room temp, on a 20-mL Heparin column (GE Healthcare) pre-conditioned with buffer NT.A (200 mM NaCl, 5 mM CaCl_2_, 25 mM HEPES/NaOH, pH 8). Unbound material was washed off the column with buffer NT.A, and weakly bound contaminants were removed by increasing the NaCl concentration to 250 mM. The protein of interest (NT-mini or NT*-mini) was then eluted into a 5-mL Ni-IMAC HisTrap Excel column (GE Healthcare) by further increasing the NaCl concentration to 600 mM (buffer NT.B). Unbound material was washed off the Ni-IMAC column with buffer NT.A, after which the recombinant protein was eluted using buffer NT.C (200 mM NaCl, 5 mM CaCl_2_, 250 mM imidazole, 25 mM HEPES/NaOH pH 8) into a 1-mL HiTrap Heparin HP column (GE Healthcare). This column was further washed with buffer NT.A, and the bound NT construct was de-tagged by loading the column with 10 mL of a 4 µg/mL SUMO protease solution. Tag cleavage was allowed to proceed in the column overnight at 4 °C, then the cleaved tag was washed off the column with buffer NT.A. At this point, NT-mini/NT*-mini was eluted from the heparin column using buffer NT.B. The obtained material was concentrated to reach a final volume of ≤ 0.5 mL and further purified by SEC on a Superdex 200 10/300 Increase column (GE Healthcare), equilibrated with GF buffer (200 mM NaCl, 25 mM HEPES/NaOH, pH 8). Finally, the purified NT-mini/NT*-mini samples were concentrated to 1 mg/mL with a Vivaspin Turbo 10 kDa MWCO concentrator (Sartorius), flash frozen in liquid nitrogen, and stored at − 80 °C. These materials served as stock solutions for all subsequent in vitro assays and cell culture experiments.

For agrin AGR-46 constructs, the filtered medium was loaded on a 5-mL HisTrap Excel column (GE Healthcare) equilibrated with buffer AG.A (500 mM NaCl, 25 mM HEPES/NaOH, pH 8). Non-specific contaminants were removed by washing the column with buffer AG.A supplemented with 25 mM imidazole, then the elution of recombinant agrin constructs was obtained using buffer AG.B (500 mM NaCl, 250 mM imidazole, 25 mM HEPES/NaOH, pH 8). Fractions were pooled and dialyzed overnight against buffer AG.A in the presence of 25 μg/mL His-tagged TEV protease. Tag and TEV protease removal was achieved by re-loading the dialyzed sample onto the 5-mL HisTrap Excel column (GE Healthcare) and collecting the unbound tagless sample. Fractions containing pure agrin constructs as assessed by SDS-PAGE analysis underwent concentration through a Vivaspin Turbo 30 kDa MWCO concentrator (Sartorius) to reach a final volume below 0.5 mL, and further purified by SEC on a Superdex 200 10/300 Increase column (GE Healthcare) equilibrated with buffer GF. All samples eluted as single peaks distant from the column void volume. Pooled fractions were concentrated to 3–10 mg/mL, flash frozen in liquid nitrogen, and stored at − 80 °C until usage.

The purification of the longer agrin AGR-124 substrate was carried out following the protocol described for the shorter AGR-46 constructs, with slight modifications in the steps following IMAC purification. Specifically, following the O/N tag-removal dialysis step, the protein was diluted 1:1 with 25 mM HEPES/NaOH pH 8, to reduce the [NaCl] to 250 mM, before applying the tagless material onto a 1-mL HiTrap Heparin HP column (GE Healthcare). The TEV protease and purification tag were then washed off the column with buffer AG.C (200 mM NaCl, 25 mM HEPES/NaOH, pH 8), while the bound AGR-124 substrate was eluted with buffer AG.A. This protein was further purified by SEC on a Superdex 200 10/300 Increase column (GE Healthcare) equilibrated with buffer AG.A. The recovered protein was concentrated (Vivaspin Turbo 30 kDa MWCO (Sartorius)) to 1.5 mg/mL and stored until usage as described previously.

### Activity Assays with Peptide Substrates

NT-mini biochemical characterization was performed spectrophotometrically, at a fixed enzyme concentration of 1 µM, with p-nitroaniline (pNa)–bearing peptide substrates (Sup. Table 2) allowing to follow product generation as an increase in absorbance at 405 nm. Initial reaction velocities (*V*_0_) extrapolated from the linear regions of those plots were used to investigate reaction parameters, compare enzymatic activities, and determine kinetics. Time-course measurements were performed at 37 °C in a clear-bottom 386-well plate (Greiner) in GF buffer. For each measurement, the final reaction volume was 20 µL. Substrate and CaCl_2_ stock solutions were prepared, respectively, as 100 mM and 1 M stocks in GF buffer (200 mM NaCl, 25 mM HEPES/NaOH, pH 8) and diluted as necessary. Stocks of BaCl_2_ and ZnCl_2_ for the corresponding experiments were prepared in an identical fashion as those of CaCl_2_. Heparin stocks were prepared as a 1 mM solution by directly dissolving heparin sodium salt (Sigma #H3393) in reaction buffer. For experiments assessing activity in relation to ionic strength, the NaCl concentration of the reaction buffer was altered to cover a 100–500 mM range; reaction parameters were otherwise unchanged from reference conditions. Reference reactions were performed using 5 mM CaCl_2_ and 1 mM peptide substrate, respectively. Each reaction was prepared on ice, with 1 mg/mL NT-mini as the last component, and briefly mixed by pipetting before measurement. To prevent evaporation, each sample was then overlayed with a small drop (~ 7 µL) of paraffin oil. Triplicate kinetics measurements were performed using CLARIOstar (BMG LABTECH) or Glomax Discovery (Promega) plate readers over the span of 230 min monitoring light absorbance at wavelengths of 405 and 600 nm. Measurements at 600 nm were used to exclude possible aggregation phenomena and correct baselines. Absorbance values were converted to concentration curves by accounting for path length and using the pNa extinction coefficient at 405 nm (9500 M^−1^ cm^−1^). The linear region of these plots (50 points in the 10–50 min measurement interval) was used to extrapolate initial reaction velocities (*V*_0_) via linear regression. The *V*_0_ values were directly fit to the Michaelis–Menten equation using Prism 6.01 (GraphPad), which provided values of apparent *k*_cat_ and *K*_m_ along with their associated errors. Propagation of statistical error value during the calculation of *k*_cat_/*K*_m_ values was carried out as described [29]. Absence of proteolytic activity in NT*-mini was assessed in the same manner. The determination of statistical significance for *V*_0_ comparisons in the presence of bivalent metal ions (Fig. 3b) or heparin (Fig. 4a) was performed with a one-way ANOVA coupled to a multiple comparison test (Bonferroni) in Prism 6.01 (GraphPad).

Assays investigating the effect of pH on peptide processing were carried out as described above, but with minor modifications in sample preparation. A series of 10 × reaction buffer stock solutions (2 M NaCl + 500 mM buffering agent) were prepared to cover a range of pH values (5.0–10.5). Sodium citrate was used to cover pH 5.0, Bis–Tris was used to cover pH 6.0–6.5–7.0, HEPES/NaOH was used to cover pH 7.5–8.0, tricine was used for pH 8.5, glycine was used for pH 9.0–9.5, and CAPS covered pH 10.0–10.5. Each experiment was prepared using 2 μL 10 × reaction buffer, 1 μL of 100 mM CaCl_2_, and 2 μL of 10 mM β-peptide substrate. Sample volume was brought up to 19 μL with water, and 1 μL NT-mini (final conc. 1 µM) was added as the last component. Substrate and CaCl_2_ stocks were prepared in water as opposed to the previously indicated buffer while NT-mini stocks remained unaltered. Mixes were prepared on ice, and kinetics measurements and *V*_0_ extrapolations were performed as described above.

Experiments investigating the role of Ca^2+^ in NT-mini activity were carried out using the 6-mer β-peptide. Substrate concentration was fixed at 10 mM and kinetics measurements were carried out with 1 µM NT-mini in the presence of increasing [Ca^2+^]. For these experiments, NT-mini was pre-diluted (1:1) with a 20 mM EDTA in GF buffer solution to allow for unambiguous evaluation of activity at low [Ca^2+^] (≤ 500 µM). CaCl_2_ solutions for these experiments were prepared as 10 × stocks (with respect to the desired assay [Ca^2+^]) adjusted to compensate for the EDTA supplemented to NT-mini. Reaction samples were prepared as described previously with slight modifications to keep NT-mini and substrate concentrations unaltered. Typical reaction mixes (20 µL) were composed as follows: 2 µL NT-mini/EDTA, 2 µL 10 × CaCl_2_, 2 µL 100 mM 6-mer β-peptide, 14 µL GF buffer. All reagents were prepared in GF buffer. Assays were monitored as described previously, and *V*_0_ measurements were used to generate a Michaelis–Menten plot for [Ca^2+^] which was used to derive the pertinent kinetic constants *k*_cat_ and *K*_m_.

### Evaluation of the Glycosylation of Recombinant Agrin Fragments

The amino acid sequences of AGR-124 and its y and z splice variants were subject to evaluation using the NetNGlyc [30] and NetOGlyc [31] servers for the in silico prediction of possible N- and O-linked glycosylation sites, respectively.

To experimentally probe the predictions, recombinant AGR-46 and AGR-124 constructs were subject to treatment with peptide N-glycosydase F (PNGase-F, New England BioLabs) for removal of N-linked glycosylation. Briefly, Glycoprotein Denaturing Buffer (New England BioLabs) was added to protein extracts to reach a 1 × concentration and the proteins were denatured at 95 °C for 10 min. After cooling, glycobuffer 2 (New England BioLabs), 1% NP-40, and 0.2 μL (100 Units) of recombinant PNGase-F were added and incubated at 37 °C for 1 h. The results were then analyzed using SDS-PAGE through comparison with non-treated samples.

O-linked glycosylations were assessed by determining the overall glycosylation content of AGR-124 using size exclusion chromatography coupled to multi-angle light scattering (SEC-MALS). Briefly, samples were loaded onto a Protein KW.802.5 (Shodex) column mounted on a Prominence high-pressure liquid chromatography (HPLC) system (Shimadzu) connected to a miniDAWN MALS detector (Wyatt Technologies), a differential refractive index detector (Shimadzu RID-20A) for quantitation of the total mass and to a UV detector (Shimadzu SPD-20A) for evaluation of the sole protein content. SEC-MALS runs were carried out at a flow rate of 1 mL/min in 50 mM TRIS/HCl, 500 mM NaCl, and pH 8.0. Results were analyzed using the protein conjugate module of the Astra software (Wyatt Technologies), using an estimated *dn*/*dc* value of 0.185 ml/g for proteins and 0.140 ml/g for glycans, and an extinction coefficient of 71,500 M^−1^ cm^−1^ for the AGR-124 sample. The calibration of the instrument was verified by injection of 10 µl of 3 mg/l monomeric BSA (Sigma-Aldrich).

### Time Course Digestions of Agrin-Like Substrates

Catalytic competence on native agrin-like substrates was assessed via time course assays using purified recombinant human agrin AGR-46 constructs. Digestions with NT-mini at 5 µg/mL were performed at a fixed starting substrate concentration of 0.5 mg/mL in GF buffer supplemented with 5 mM CaCl_2_. All necessary dilutions were performed with the reference reaction buffer. Typical reaction mixes were prepared on ice in a PCR tube (total reaction volume 110 μL) and were composed of 500 μg/mL agrin construct, 5 mM CaCl_2_, and 5 µg/mL NT-mini. NT-mini was added last, and then the solution was mixed by gentle pipetting. Reaction mixes were maintained at 37 °C in a PCR thermocycler (Eppendorf) and 10 µL samples were collected after 0, 5, 10, 15, 20, 25, 30, 60, 90, and 120 min. Each sample was immediately supplemented with 5 µL of SDS-PAGE reducing sample buffer, boiled for 10 min at 95 °C to block the reaction, and loaded on a 16% polyacrylamide gel. Protein bands were stained with colloidal Coomassie blue solution (0.02% Brilliant Blue G250 *w*/*v*, 20% ethanol *v*/*v*, 5% Al_2_(SO_4_)_3_ *w*/*v*, 1.05 M phosphoric acid) overnight and carefully destained with 10% acetic acid *v*/*v* and 20% ethanol *v*/*v*. Images for analysis were taken with a ChemiDoc imager (BioRad) and substrate/product band densities were measured with ImageJ [32]. The intensity values for the substrate bands obtained at 0 min of digestion were set to represent starting substrate concentration (0.5 mg/mL or 10 µM), and the normalized intensities associated with subsequent time point bands were then plotted as a function of time. Prism6 (Graphpad) was used to plot a non-linear regression curve from which pseudo-first-order rate constants for substrate consumption (*k*_s_) and product generation (*k*_CAF-22_) were derived, along with their associated errors. The same procedure was followed for time-course digestions of the agrin-like AGR-124 substrate, and for experiments with NT*-mini. For AGR-124, the polyacrylamide gel composition (6%) was adjusted to maximize the resolution of high MW CAF-110 and CAF-90 product bands. Statistical significance was determined using a one-way ANOVA coupled with a multiple comparison test (Bonferroni) in Prism 6.01 (GraphPad) to compare *k*_s_ values of AGR-46 y0z0 with AGR-46 y4z0, AGR-46 y0z0 with all y0z + variants, and AGR-46 y4z0 with all y4z + variants.

### Thermal Denaturation Assays

The effects of Ca^2+^, Zn^2+^, Ba^2+^, heparin, EGTA, or EDTA on NT-mini’s stability were assayed directly using thermal denaturation coupled to nano-differential scanning fluorimetry (nano-DSF). For these experiments, the concentration of NT-mini was fixed at 2 µM (0.1 mg/mL), while bivalent metal ions (Ca^2+^, Zn^2+^, Ba^2+^) were assayed at 5 mM, chelators (EGTA, EDTA) were tested at 10 mM, and heparin was used at 1 mM. All chemicals (Sigma-Aldrich) were dissolved directly in GF buffer, to prepare stock solutions at high concentrations: CaCl_2_ 1 M, BaCl_2_ 1 M, ZnCl_2_ 1 M, NT-mini 20 µM (≈ 1 mg/mL), heparin (Sigma #H3393) 4 mM (≈ 72 mg/mL), EGTA 1 M, and EDTA 1 M. The same buffer was also used for all necessary dilutions to reach the assay concentrations from those stocks.

For the evaluation of Ca^2+^-induced NT-mini stabilization, thermal denaturation nano-DSF experiments were conducted in the presence of increasing [Ca^2+^] covering the following concentrations: 1 µM, 10 µM, 100 µM, 1 mM, 10 mM, 100 mM, 1 M, 2 M. NT-mini was pre-treated with EDTA (10 mM) before being assayed at a final conc. of 2 µM. CaCl_2_ solutions were prepared as 10 × stocks (with respect to the desired assay conc.) adjusted to compensate for the EGTA supplemented with NT-mini. All stock solutions and necessary dilutions were prepared in GF buffer (200 mM NaCl, 25 mM HEPES/NaOH, pH 8).

Protein stability at differing pH values was tested at the same final 2 μM NT-mini concentration. Samples were prepared with the same 10 × reaction buffers used for the peptide digestion assays to cover the same pH range. Similarly, in these experiments, the necessary buffer dilutions were performed with water.

Protein unfolding was monitored in Tycho quartz capillaries (Nanotemper) using a Tycho NT.6 instrument (Nanotemper), which allowed for the determination of NT-mini’s unfolding temperatures (*T*_i_) in the assayed conditions. The smoothed data were re-plotted using Prism 6.01 (Graphpad) for comparative purposes. *T*_i_ values for increasing Ca^2+^-induced stabilization were plotted as a function of [Ca^2+^] and used to extrapolate an *EC*_*50*_ via sigmoidal 4PL non-linear regression in Prism 6.01 (Graphpad) with default parameters.

### SPR Determination of Heparin Binding and Affinity

Measurements of heparin binding were performed in a Biacore T200 instrument (GE Healthcare) using a heparin-coated chip (Xantec). All analyses were performed in tris-buffer saline (TBS, 150 mM NaCl, 50 mM Tris–HCl, pH 7.5) unless otherwise noted. After each sample injection, the chip was regenerated using a TBS solution supplemented with 1 M NaCl. For the identification of the heparin-binding domain, the murine SRCR3 (human SRCR4) domain and NT-mini were run individually at a concentration of 50 nM with a contact time of 60 s at a flow of 50 µL/min. For the estimation of NT-mini’s affinity for heparin, a decreasing series of 9 NT-mini concentrations (200–0.78 nM) were obtained by twofold serial dilution in TBS, starting from a protein stock of 22 µM. Steady-state binding was assayed using the single-cycle kinetics mode with a contact time of 60 s and a flow rate of 30 µL/min. Curves were analyzed using the kinetics fit of the Biacore evaluation software (GE Healthcare) with the Rmax parameters set to local fit. For visualization purposes, the SPR traces were exported and re-plotted using Prism 6.01 (Graphpad).

### C2C12 Cultures

For maintenance, C2C12 myoblasts (kindly provided by DM Rossi, ICS Maugeri, Pavia) were grown in 75 cm^2^ T-flasks (VWR) at 37 °C in a humidified 5% CO_2_ atmosphere. Cells were maintained in a high-glucose DMEM (Thermo Fisher Scientific) medium supplemented with 10% (*v*/*v*) fetal bovine serum (FBS, Thermo Fisher Scientific) and 1% (*v*/*v*) non-essential amino acids (NEAA, Thermo Fisher Scientific). Growth medium was refreshed on average every 48 h, and cells were generally split 1/10 when at 60–70% confluence.

To perform differentiation experiments, cells were transferred to 6-well plates containing sterilized glass cover slips and seeded at ~ 50% confluence. Cultures were allowed to stabilize for 24 h before inducing differentiation. This was obtained using high-glucose DMEM medium with a reduced FBS content (1%) but still supplemented with 2% NEAA. Differentiation was generally protracted for 7 days before proceeding with immuno-staining. Growth media was refreshed every 48 h.

Treatments with NT-mini/NT*-mini (50 ng/mL) were performed throughout the 7-day differentiation period by supplementing the differentiation medium with the purified recombinant protein. All necessary stock dilutions were performed directly in differentiation medium.

### C2C12 Staining and Analysis

Evaluation of myoblast-to-myotube differentiation was carried out using a live stain protocol adapted from the procedures described in Stanga et al. [33], McMorran et al. [34], and Harrison et al. [35]. Briefly, cellular boundaries were identified by staining cell-surface glycans with an Alexa645-wheat germ agglutinin (WGA) conjugate (Thermo Fisher Scientific), while nuclei were stained using Hoechst dye (Sigma-Aldrich). Acetylcholine receptor (AChR) clustering was evaluated using an Alexa594-bungarotoxin (Btx) conjugate (Thermo Fisher Scientific). Hoechst and Btx stains were added directly to the media in the 6-well plate, in a 1/2000 (*v/v*) and 1/1000 (v/v) ratio respectively, and incubated at 37 °C for 45 min. Staining with WGA was also performed directly in the culture media, but in a 1/250 (v/v) ratio and with a 15 min incubation time. After staining, the cells were washed once with PBS, fixed with a 4% (*w*/*v*) paraformaldehyde (PFA) in PBS solution at room temperature for 15 min, and washed again with PBS. Finally, the cover slips were mounted on microscopy slides with a drop of mounting media (Sigma-Aldrich). These mounts were wrapped in tin foil and allowed to set O/N at 4 °C before being imaged using a SP8 white-laser confocal microscope (Leica Microsystems). Filters were adjusted specifically for each stain to visualize nuclei (Hoechst, Ex 405 nm, Em 429 nm), AChRs (Btx, Ex 598 nm, Em 634 nm), and cell-surface glycans (WGA, Ex 653 nm, Em 692 nm). Z-stacks of 0.7-µm thickness were collected at 40 × magnification, and images were analyzed with ImageJ [32]. Myotube identification and assignment of nuclei were performed by manual slice-by-slice assessment of the Z-stacks. Fusion indexes were calculated as the fraction of total nuclei incorporated into myotubes. These data were normalized to the non-treated control conditions and analyzed on Prism 6.01 (GraphPad) to assess the statistical significance of the observed differences with a one-way ANOVA coupled with a multiple comparison test (Bonferroni). Violin-plots of analyzed data were generated with MATLAB [36] using the “Violinplot-Matlab” extension [37].

### Electrophysiology in Acute Hippocampal Slices

Animal maintenance and experimental procedures were performed according to the international guidelines of the European Union Directive 2010/63/EU on the ethical use of animals and were approved by the local ethical committee of the University of Pavia (Italy) and by the Italian Ministry of Health (628/2017-PR). Acute 300-µm-thick brain slices were cut on the coronal plane from the hippocampus of 20–23-day-old C57BL/6 mice (either sex), as reported previously [38]. Briefly, mice were deeply anesthetized with halothane (Sigma-Aldrich) and killed by decapitation to remove the hippocampus (either left or right) for acute slice preparation using a vibratome (VT1200S, Leica Microsystems). The cutting procedure was performed in an ice-cold solution containing 87 mM NaCl, 2.5 mM KCl, 25 mM NaHCO_3_, 1.25 mM NaH_2_PO_4_, 75 mM sucrose, 25 mM glucose, 0.2 mM CaCl_2_, and 7 mM MgCl_2_ and bubbled with 95% O_2_ and 5% CO_2_ (pH 7.4). Slices were recovered at 32 °C for 1 h in Krebs solution containing 120 mM NaCl, 2 mM KCl, 1.2 mM MgSO_4_, 26 mM NaHCO_3_, 1.2 mM KH_2_PO_4_, 2 mM CaCl_2_, and 11 mM glucose, equilibrated with 95% O_2_–5% CO_2_ (pH 7.4). Subsets of slices were incubated for 90 min in Krebs solution containing either NT-mini or NT*-mini (100 ng/ml, in both cases). The slices were then transferred to the recording chamber of an upright microscope (Zeiss), perfused with oxygenated Krebs solution (2 ml/min), and maintained at 32 °C using a Peltier feedback device (Warner Instruments). Krebs solution contained NT-mini or NT*-mini (100 ng/ml, in both cases) in the subset of recordings on slices pre-incubated with the corresponding enzyme. Whole-cell patch-clamp recordings were performed on hippocampal CA1 pyramidal neurons using glass borosilicate patch pipettes (resistance 5.5–7 MΩ) filled with an intracellular solution containing (mM) 138 mM K-gluconate, 8 mM KCl, 10 mM HEPES, 0.5 mM EGTA, 4 mM Mg-ATP, and 0.3 mM Na-GTP adjusted to pH 7.3 with KOH. Signals were acquired using a Multiclamp 700B amplifier (Molecular Devices; cutoff frequency of 10 kHz) and digitized with a Digidata 1440A interface (Molecular Devices). After reaching the whole-cell configuration, voltage steps were used (from − 70 mV, 10 mV per step) to elicit the Na current and to evaluate passive properties. Neuronal intrinsic excitability was assessed in current-clamp mode from resting membrane potential (Vrest), by injecting 1 s current steps of increasing amplitude (from − 50 pA to 400 pA, with 25 pA increment). Membrane capacitance (Cm), input resistance (Ri), and series resistance (Rs) were calculated as reported previously [38, 39]. No significant difference was found in these parameters among the different conditions (controls, NT-mini-treated and NT*-mini treated neurons). Signals were analyzed offline using Clampfit 10 (pClamp suite, Molecular Devices). All drugs were obtained from Sigma-Aldrich (Merck). Data are reported as mean ± MSE (standard error of the mean) and compared for statistical significance using unpaired Student’s *t* test.

## Results

### Production and Purification of NT-Mini

In line with previously published data [16], production of full-length, enzymatically active human NT proved unfeasible, thus requiring the engineering of an extensive library of shorter variants derived from the original cDNA. Of these, a single construct termed NT-mini (Gly497-Leu875; Fig. 1a), bearing the catalytically active SP domain, was successfully produced in a HEK293 mammalian cell suspension system [28]. Purification of NT-mini from the cell culture media was obtained with a “mixed-mode” affinity chromatography approach based on immobilized metal ion (IMAC) and heparin affinity. To avoid the partial zymogen-enzyme activation reported for the full murine NT [16], the activation process was enhanced by co-transfecting cells with cDNA for both NT-mini and the pro-protein convertase Furin [17, 40]. The final result was a highly pure and homogeneous sample almost entirely present in the active two-chain enzyme conformation (Sup. Fig. 1), with an estimated yield of 1 mg per L of cell suspension culture.

The same approach allowed for the production and purification of the inactivated-by-design Ser825Ala variant of NT-mini (NT*-mini; Fig. 1a). During production and purification, this mutant behaved like its wild-type (wt) counterpart, and no significant differences in yield or construct stability were observed.

To determine NT-mini’s catalytic competence, its activity was investigated using synthetic chromogenic peptides and recombinantly produced agrin constructs.

### NT-Mini Cleaves Synthetic β-Peptides with High Specificity

Biochemical characterization of NT-mini was initially carried out using synthetic 6-mer peptides bearing a C-terminal chromogenic p-nitroaniline (pNa) moiety mimicking agrin’s α- (α-peptide) or β- (β-peptide) cleavage sites. Proteolytic processing was monitored over time by following pNa release at 405 nm. The resulting kinetics curves, analyzed over a 1–20 mM substrate range, evidenced barely detectable processing for the α-peptide (Sup. Fig. 2; Table 1), and better processing of the β-peptide, resulting in catalytic efficiency (calculated as *k*_cat_/*K*_m_) of 7.81 ± 1.56 × 10^−6^ M^−1^ s^−1^ (Fig. 1b; Table 1).

**Table 1:** Cleavage of peptide substrates by NT-mini

Substrate	*V*_0_ × 10^−6^ (mM s^−1^)	Activity (%)	Difference (%)	*V*_max_ × 10^−6^ (mM s^−1^)	*K*_m_ (mM)	*k*_cat_ × 10^−2^ (s^−1^)	*k*_cat_*/K*_m_ × 10^−6^ (M^−1^ s^−1^)
β-peptide	7.83 ± 0.63	100.00 ± 11.45	0.00 ± 16.19	79.53 ± 5.39	6.16 ± 1.16	4.81 ± 0.33	7.81 ± 1.56
β-(5 mer)	8.39 ± 0.59	107.75 ± 11.56	7.57 ± 16.27	217.98 ± 30.45	17.25 ± 3.92	17.42 ± 2.43	10.10 ± 2.69
β-(4 mer)	4.55 ± 0.23	58.42 ± 5.54	-41.58 ± 12.72	80.72 ± 3.22	17.19 ± 1.13	6.45 ± 0.26	3.75 ± 0.29
β-(3 mer)	2.86 ± 0.12	36.75 ± 3.37	-63.25 ± 11.94	107.25 ± 9.56	42.44 ± 4.86	8.57 ± 0.76	2.02 ± 0.29
Lys-pNa	0.23 ± 0.09	2.99 ± 1.19	-97.01 ± 11.51	ND	ND	ND	ND
α-peptide	1.02 ± 0.07	13.13 ± 1.43	-86.87 ± 11.54	ND	ND	ND	ND

The absence of several NT accessory domains from NT-mini raised the question as to whether this construct might have lost the native protein’s high selectivity. We therefore assayed NT activity on a small library of shorter synthetic β-peptides (5–3 mers) as well as individual amino acids conjugated with pNa. Respective *V*_*0*_s at a standardized substrate concentration (1 mM) were plotted against the reference (6-mer) β-peptide (Sup. Fig. 2) highlighting a substrate-dependent response from NT-mini. While the shortest peptides (4, 3, 1-mer) were processed progressively less efficiently (− 41.58 ± 12.72%, − 63.25 ± 11.94%, and − 97.01 ± 11.51%, respectively), the 5-mer β-peptide was processed as efficiently as its “parent” 6-mer peptide (+ 7.75 ± 16.27%). The cleavage of these different β-peptide substrates was further investigated by evaluating the Michaelis–Menten kinetics parameters for each peptide (Fig. 1b; Table 2). This confirmed the initial observations and highlighted the strong preference for the longer 5-mer and 6-mer peptides, which were processed with similar efficiency (*k*_cat_/*K*_m_ 10.10 ± 2.69 × 10^−6^ and 7.81 ± 1.56 × 10^−6^ M^−1^ s^−1^, respectively). *K*_m_ estimates for each substrate seemed to highlight a reduction in affinity for progressively shorter peptides, a trend which was not reflected in the *V*_max_ values (Table 1). These show a peak of activity in correspondence to the 5-mer peptide (217.98 ± 30.54 × 10^−6^ mM s^−1^), whose *V*_max_ was estimated to be 2- to threefold higher than all other substrates. While this appears in contrast with the initial *V*_0_’s measured at 1 mM substrate, it is likely that, given the markedly low reaction velocities (Fig. 1b), the *V*_max_ values were overestimated (and *K*_m_’s underestimated). Differently, when comparing profiles on the same plot (Fig. 1b), it is likely that the 5-mer’s-extrapolated *V*_max_ is reflective of an effectively increased reaction velocity as compared to the 6-mer peptide. This might have been masked in initial *V*_0_ measurements by rate-limiting substrate concentrations given the overall low affinity (mM) of NT-mini (or NT) for short synthetic peptide substrates [17586728].

**Table 2:** Cleavage rates of agrin-like substrates by NT-mini

Substrate	k_S_ (× 10^−4^ s^−1^)	SD
y0z0	12.33	± 1.82
y0z8	8.68	± 0.56
y0z11	4.63	± 0.45
y0z19	5.72	± 0.21
y4z0	15.14	± 1.27
y4z8	9.25	± 0.88
y4z11	5.99	± 0.42
y4z19	7.02	± 0.55

### pH-Dependent Modulation of NT-Mini Enzymatic Activity

The same β-peptide that allowed the characterization of NT-mini’s proteolytic activity was used to assess its pH dependency. The initial reaction velocity of NT-mini was evaluated across a range of pH at a fixed β-peptide (1 mM) concentration, allowing to express activity as a function of pH (Fig. 1c, blue line). This revealed that NT-mini’s peak activity was restrained to a relatively narrow pH range (8.0–9.0), slightly higher than the physiological reference value of 7.5. The increasing acidification of reaction conditions from pH 8.0 to 5.0 correlated to a progressive reduction of enzymatic activity, with near complete loss of activity at pH ≤ 6.0. Likewise, values above pH 9.0 induced a sharp drop in NT-mini’s activity, with minimal *V*_0_ values recorded at pH ≥ 10.0.

To correlate variations in enzymatic activity with alterations in protein folding and stability, a series of nano-differential scanning fluorimetry (DSF) thermal denaturation assays were performed to cover the same pH range seen to influence protein activity (Fig. 1c, dashed red line). NT-mini was seen to be most stable in acidic to mildly acidic conditions (*T*_i_ 66 °C, pH ≤ 6.5), while being progressively destabilized with increasing pH (*T*_i_ 51.8 °C, pH 10.5). This highlighted how enzymatic activity is likely affected by the loss of folding stability in alkaline conditions (pH > 9.0), while inactivation in acidic environments (pH < 6.0) is more likely dependent on the protonation of amino acid residues critical for substrate recognition or catalysis.

### NT-Mini Cleaves Agrin-Like β Substrates

**Fig. 2:**
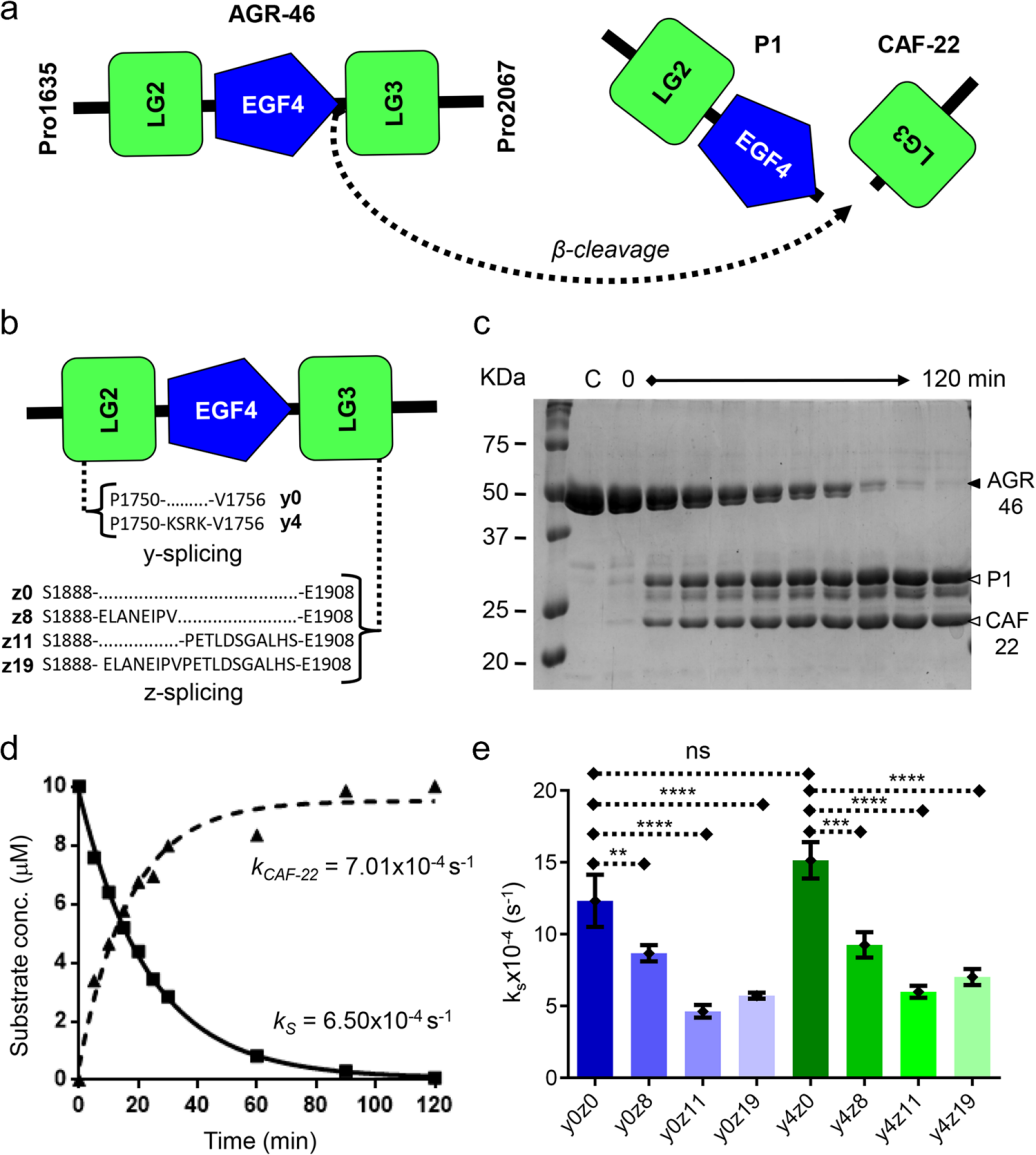
Cleavage of agrin-like substrates by NT-mini. **a** Diagram depicting cleavage of agrin-like AGR-46 substrates by NT-mini generating two smaller products: the LG2-EGF4 cluster (P1), and the LG3 domain (CAF-22). **b** Representation of y and z splicing inserts on AGR-46 substrates. Numbering refers to human agrin (O00468-1). **c** Representative SDS-PAGE of a time-course digestion of agrin-like substrates with NT-mini. The substrate, AGR-46, is processed over time (120 min total) to generate digestion products, P1 and CAF-22. Substrate consumption and product generation are assessed by densiometric analysis. **d** Representative time-resolved activity plot of product consumption and substrate generation. Curves are obtained via non-linear regression with single-phase decay (substrate) or association (product) models in Prism 6.01. These provide corresponding pseudo-first-order rate constants for substrate consumption (*k*_*S*_) and product generation (*k*_*CAF-22*_). **e** Comparison of *k*_S_ activity rates of NT-mini on agrin-like substrates covering all splice variant combinations. Data represents the mean from measurements of triplicate independent experiments (*n* = 3) and error bars correspond to their standard deviations (SD). ns, not significant; **p ≤ 0.01; ***p ≤ 0.001; ****p ≤ 0.0001

The initial assays with synthetic peptides allowed us to investigate NT-mini’s activity and observe catalytic competence on substrates bearing the β-cleavage site sequence. However, short peptides are a very simplified representation of the highly glycosylated, multi-domain agrin substrate, especially when considering agrin’s multiple splice variants [26, 27]. Therefore, we decided to evaluate whether NT-mini retained the ability to cleave native-like agrin substrates. To this end, we generated two families of engineered human agrin C-terminal constructs to be used as substrates in time-course digestion experiments. The first (AGR-46) limited to the last three LG-EGF-LG domains (Pro1635-Pro2068) (Fig. 2a) was developed to address β-site cleavage while covering all possible combinations of agrin’s y and z splicing insertion sites (Fig. 2b), and the second (AGR-124) spanning agrin’s 9th FS domain to its last LG domain (Ala893-Pro2068) (Sup. Fig. 3a) was designed to include both cleavage sites. Considering the high degree of glycosylation of native human agrin [41–44], we performed a series of quality control experiments to confirm the presence and the amount of glycosylations of our engineered agrin C-terminal constructs. Firstly, we ran in silico predictions for N- and O-linked glycans on the AGR-124 amino acid sequence (Sup. Fig. 4a) and found that the short C-terminal AGR-46 construct should bear no N-linked glycosylations, whereas only one is predicted for the longer AGR-124 construct. Regarding O-linked glycosylations, as expected, we found a complex pattern indicating multiple sites subject to modification; no additional changes were expected to occur in the presence of splice insertions. To experimentally validate these predictions, we treated both recombinant AGR-124 and AGR-46 samples with peptide N-glycosydase-F (PNGAse-F) and found that only the AGR-124 displays minimal shift after PNGAse-F treatment, consistent with limited N-linked glycosylations (Sup. Fig. 4b). We then performed size exclusion chromatography coupled to multi-angle light scattering (SEC-MALS) analysis of recombinant AGR-124, observing heterogeneous protein oligomers consistently decorated with glycans (presumably O-linked) contributing around 10% of the total mass of the sample (Sup. Fig. 4c). Time-course digestions of these substrates were analyzed with SDS-PAGE, separating substrates (AGR-46 or AGR-124) from intermediate (CAF-110) and final digestion products (CAF-90, P1, and CAF-22), to generate densitometric time-resolved activity plots (Fig. 2c; Sup. Fig. 3b). Pseudo-first-order rate constants for AGR-46 substrate consumption (*k*_S_) and LG3 (CAF-22) product formation (*k*_CAF-22_) were estimated with non-linear regression one-phase decay/association models (Fig. 2d). The *k*_S_ values were used to compare the relative activity observed for the different AGR-46 y and z splice insert combinations (Fig. 2e).

NT-mini displayed catalytic competence for all assayed agrin-like substrates and was found capable of both α- (Sup. Fig. 3) and β-site cleavage (Fig. 2c). AGR-46 splice variants were processed with *k*_S_ values ranging from 4.63 × 10^−4^ to 15.14 × 10^−4^ s^−1^ (Table 2). A substrate-dependent modulation of enzymatic activity was observed for the different splice variants, loosely correlating a reduction in activity with the presence of longer z inserts. Agrin-like substrates without any z insert were among those best processed, whereas the lowest levels of activity were observed for substrates with the z11 and z19 inserts. The y4 insertion initially seemed to correlate with the highest activity; however, this positive effect did not appear to be statistically significant when comparing the y4z0 and y0z0 variants (Fig. 2e; *p* > 0.05). Additionally, any appreciable difference was lost with the addition of any z insert, and y4z+ substrates were processed at rates generally comparable to their y0 counterparts (Fig. 2e).

The NT*-mini construct was confirmed as inactive with the same procedures used to investigate the activity of the catalytically competent NT-mini. As expected, the Ser825Ala mutation successfully abolished NT-mini’s proteolytic activity (Sup. Fig. 5a–b), and NT*-mini was unable to process both the agrin-like AGR-46 substrates (Sup. Fig. 5a) and the synthetic β-peptides (Sup. Fig. 5b).

### Modulation of Catalytic Activity by Metal Ions

In addition to a high degree of specificity, literature describes two key factors influencing NT activity: [Ca^2+^] [16] and heparin/heparan-sulfates [17].

**Fig. 3:**
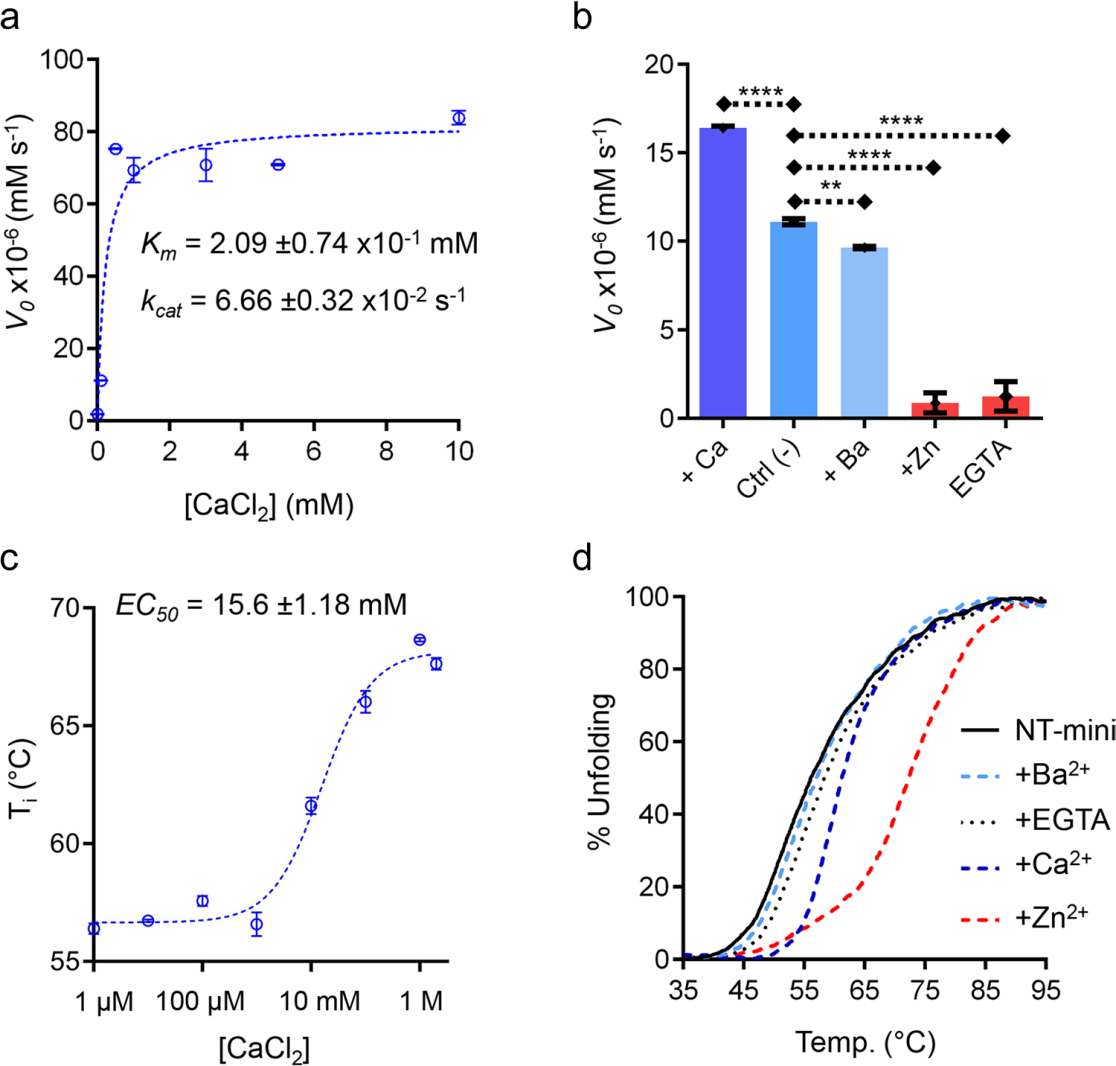
Modulation of NT-mini by bivalent metal ions. **a** Michaelis–Menten plots of NT-mini activity at fixed 6-mer β peptide concentration (10 mM) with increasing [Ca^2+^] concentrations (0–10 mM). *K*_m_ and *k*_cat_ were estimated using non-linear regression (dashed line) Michaelis–Menten enzyme-kinetics models in Prism 6.01. The absence of calcium (0 mM) was simulated with 10 mM EDTA. Single data points represent the mean of triplicate independent (*n* = 3) experiments; error bars correspond to the standard deviation (SD) of each value. **b** Comparative *V*_0_ plot of NT-mini activity at 1 mM β-peptide in presence of Ca^2+^ (5 mM), no added metal ion, BaCl_2_ (5 mM), ZnCl_2_ (0.2 mM), or EGTA (10 mM). Data is shown as the mean from data points *n* = 3 independent experiments with error bars indicating the relative SD: **, *p* ≤ 0.01; ****, *p* ≤ 0.0001. **c** Thermal stabilization of NT-mini unfolding temperature (*T*_i_) in presence of increasing [Ca^2+^] (1 μM–2 M). Line of best fit (dashed blue line) was generated with a logarithmic non-linear regression (4PL) in Prism 6.01 (GraphPad) and used to estimate the *EC*_*50*_. Data shows the averaged measurements from *n* = 3 independent experiments, and error bars correspond to their SD. **d** Normalized (to the highest and lowest values of each curve) plot of NT-mini nano-DSF thermal denaturation curves with different bivalent metal ions. The reference curve (black line), obtained in absence of any additive, is compared to curves generated by denaturation in presence of 10 mM Ba^2+^ (dashed cyan line), EGTA (dotted black line), Ca^2+^ (dashed blue line), and Zn^2+^ (dashed red line). Zn^2+^ induces the greatest degree of stabilization, Ca^2+^ has a significant but lower impact, and Ba^2+^ and EGTA have no visible effect on protein stability

To quantitatively determine if NT-mini is calcium-regulated, its catalytic activity was assessed by monitoring the processing of the 6-mer β-peptide in the presence of increasing Ca^2+^ concentrations. The substrate concentration was fixed at 10 mM, and a Michaelis–Menten plot covering a range of [Ca^2+^] up to 10 mM was generated (Fig. 3a). This evidenced a clear positive response of NT-mini’s activity in relation to [Ca^2+^], with maximal initial velocities observed using 500 µM CaCl_2_, and likely reaching saturation at higher concentrations. Extrapolating catalytic constants via Michaelis–Menten non-linear regression yielded an estimate for Ca^2+^ affinity of 209.25 ± 7.42 μM.

To evaluate the Ca^2+^ specificity of NT-mini, its enzymatic activity was assessed in the presence of other divalent metal ions and the strong Ca^2+^ chelating agent ethylene glycol tetraacetic acid (EGTA). Assays performed in the presence of 5 mM Ba^2+^ showed that barium was unable to yield the same reaction velocities from NT-mini that could be observed from identical [Ca^2+^]. Furthermore, the *V*_0_s observed using 5 mM Ba^2+^ more closely resembled the un-supplemented control than the corresponding Ca^2+^ supplemented reaction (Fig. 3b). Similarly, nano-DSF of NT-mini in the presence of incrementally higher concentrations of Ca^2+^ correlated to an increasingly stable protein (Fig. 3c), while the presence of Ba^2+^ had little influence on NT-mini’s stability (Fig. 3d). Interestingly, the calcium-induced stabilization did not reach its maximum at 500 μM Ca^2+^ but continued to increase reaching maximum stability at 1 M Ca^2+^. Analyzing the nano-DSF unfolding temperatures as a function of increasing [Ca^2+^] allowed to estimate an EC_50_ for Ca^2+^-induced NT-mini stabilization of 15.74 ± 3.41 mM (Fig. 3c). This is reminiscent of the affinities documented for Ca^2+^-binding to the murine accessory SRCR3 domain (10.1 mM) [24]. When taken together with the activity data, these observations hint at the presence of multiple calcium-binding sites on NT-mini.

Surprisingly, the addition of Zn^2+^ induced markedly different responses. While nano-DSF data highlighted a strong positive effect on the protein’s stability (Fig. 3d), this was not the case for proteolytic activity. Namely, concentrations of Zn^2+^ as low as 0.2 mM fully inactivated NT-mini, completely blocking the processing of the synthetic β-peptides (Fig. 3b) and agrin-like substrates (Fig. 3b; Sup. Figure 4c). Metal ion chelation by EGTA resulted in comparable levels of inactivation (Fig. 3b), while having no effect on protein stability (Fig. 3d), suggesting a specific catalytic role for transiently bound Ca^2+^.

**Fig. 4:**
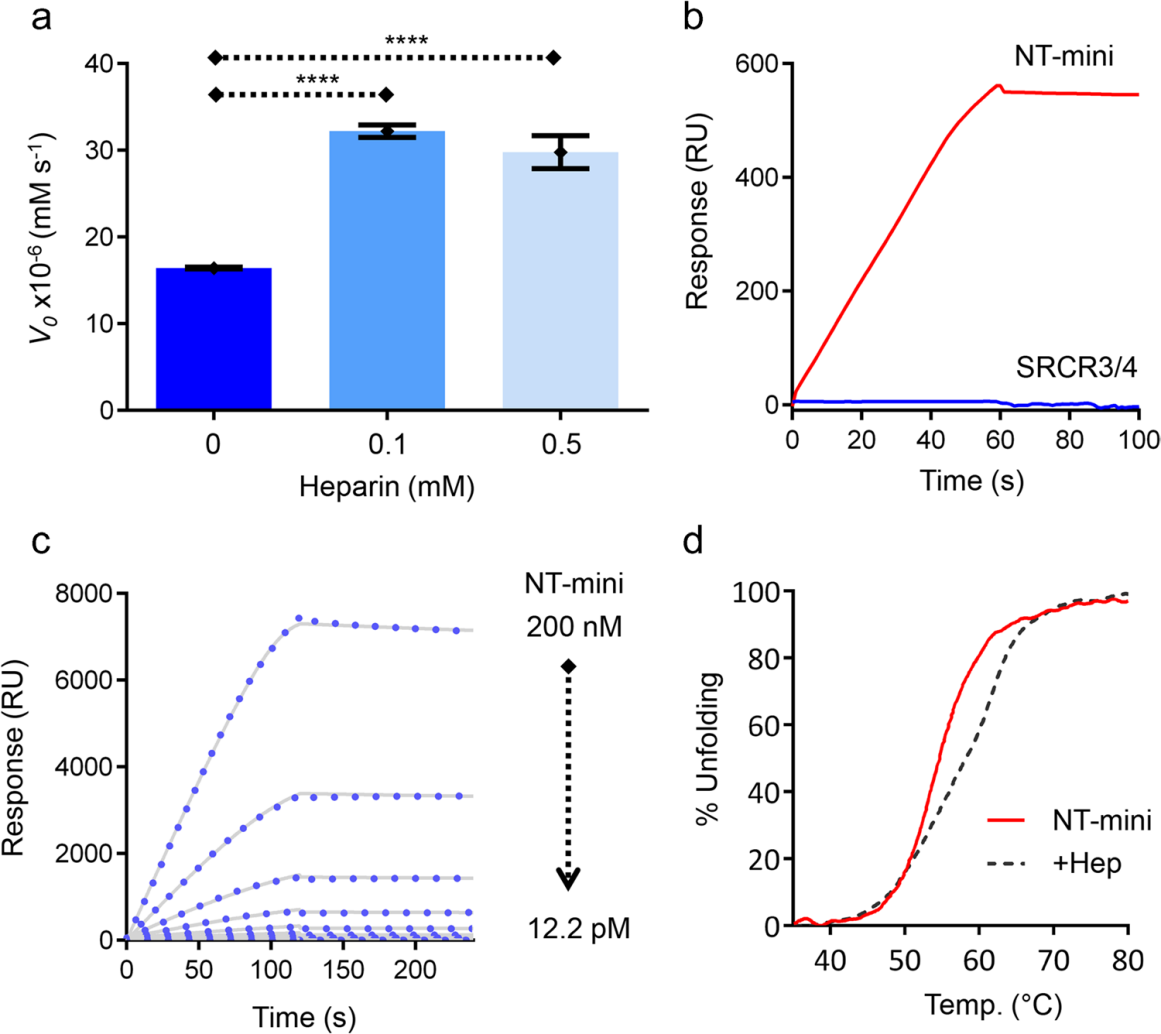
Modulation of NT-mini by heparin. **a** Activity of NT-mini in presence of heparin (0.0, 0.1, and 0.5 mM). *V*_0_s of the reactions were determined at a fixed 1 mM 6-mer β-peptide. Error bars indicate the standard deviation (SD) of averaged data points from triplicate independent (*n* = 3) experiments, ****p ≤ 0.0001. **b** Identification of the heparin-binding domain performing SPR on a heparin chip. NT-mini (red line) strongly binds the heparin chip, while the SRCR domain (blue line) preceding the SP domain does not. **c** Plot of the SPR response of increasing NT-mini concentration (12.2 pM–200 nM) on a heparin chip. Dotted lines show the recorded sensogram; grey lines represent fit kinetics curves. Curve fit was obtained with the kinetics analysis of the BiacoreT200 evaluation software with local Rmax. **d** Comparison of nano-DSF thermal unfolding of NT-mini without heparin (red line), and in presence of 1 mM heparin (dashed black line). Unfolding profiles were normalized to the highest (100%) and lowest (0%) values of each curve and used to indicate the extent of thermally denatured protein (% unfolding)

### Modulation of Activity by Heparin and Identification of the Responsible Domain

Another distinctive feature of NT is its reported interaction with heparin and heparan sulfates [17]. However, the domains responsible were not identified, meaning that this property could depend on the catalytic SP domain alone or may require one or more of the Kr/SRCR1-4 accessory domains. Therefore, NT-mini represented a unique opportunity to pinpoint the predominant heparin-binding domain.

NT-mini’s activity was tested for heparin modulation by carrying out in vitro β-peptide digestion assays in the presence or absence of heparin. Remarkably, NT-mini responded positively to heparin, and the addition of 0.1 or 0.5 mM heparin was seen to induce an approximate twofold increase in catalytic activity independent of heparin concentration (Fig. 4a). This suggests the presence a heparin-binding site in NT’s last two domains, and given the “short” nature of the 6-mer substrate used for testing, it seems to hint at a role for heparin in facilitating substrate processing by NT.

Previously [24], we reported the successful production and purification of the isolated SRCR domain included in NT-mini; here, we used both constructs to identify the NT domain responsible for heparin binding. Surface plasmon resonance (SPR) experiments using a heparin-coated chip highlighted a clear binding response for NT-mini which was absent for the SRCR domain alone (Fig. 4b). Additionally, by assaying the SPR response to NT-mini concentrations up to 200 nM, we could estimate a picomolar binding affinity (342 pM) (Fig. 4c). Interestingly, while strongly influencing activity, the high-affinity interaction between heparin and NT-mini was seen to have only a mildly positive influence on protein stability (Fig. 4d).

### NT-Mini Hampers Myotube Formation In Vitro

To investigate the impact of NT-mini in a “muscle” cellular context [11], we used C2C12 mouse myoblasts, a subclone of a well-documented cell line (C2) often used to address muscle cell development and NMJ maturation [33, 45–50]. Myoblast-to-myotube differentiation was induced by serum starvation, and parallel experiments with cells treated with either NT-mini or NT*-mini were carried out and compared with untreated controls. Fully differentiated cultures were stained to detect cell nuclei, acetylcholine receptors (AChRs), and cell-surface glycans (Sup. Fig. 6a). Cell boundaries were determined based on the glycan and AChR stains which allowed us to identify differentiated myotubes as cells containing 3 or more nuclei [33, 51, 52] (Sup. Fig. 6b–c). The effect of NT-mini’s activity on myotube formation was then quantified and cross-compared with NT*-mini and the non-treated controls. Comparisons were based on the relative fusion indexes (FI), calculated as the fraction of total nuclei incorporated in myotubes.

**Fig. 5:**
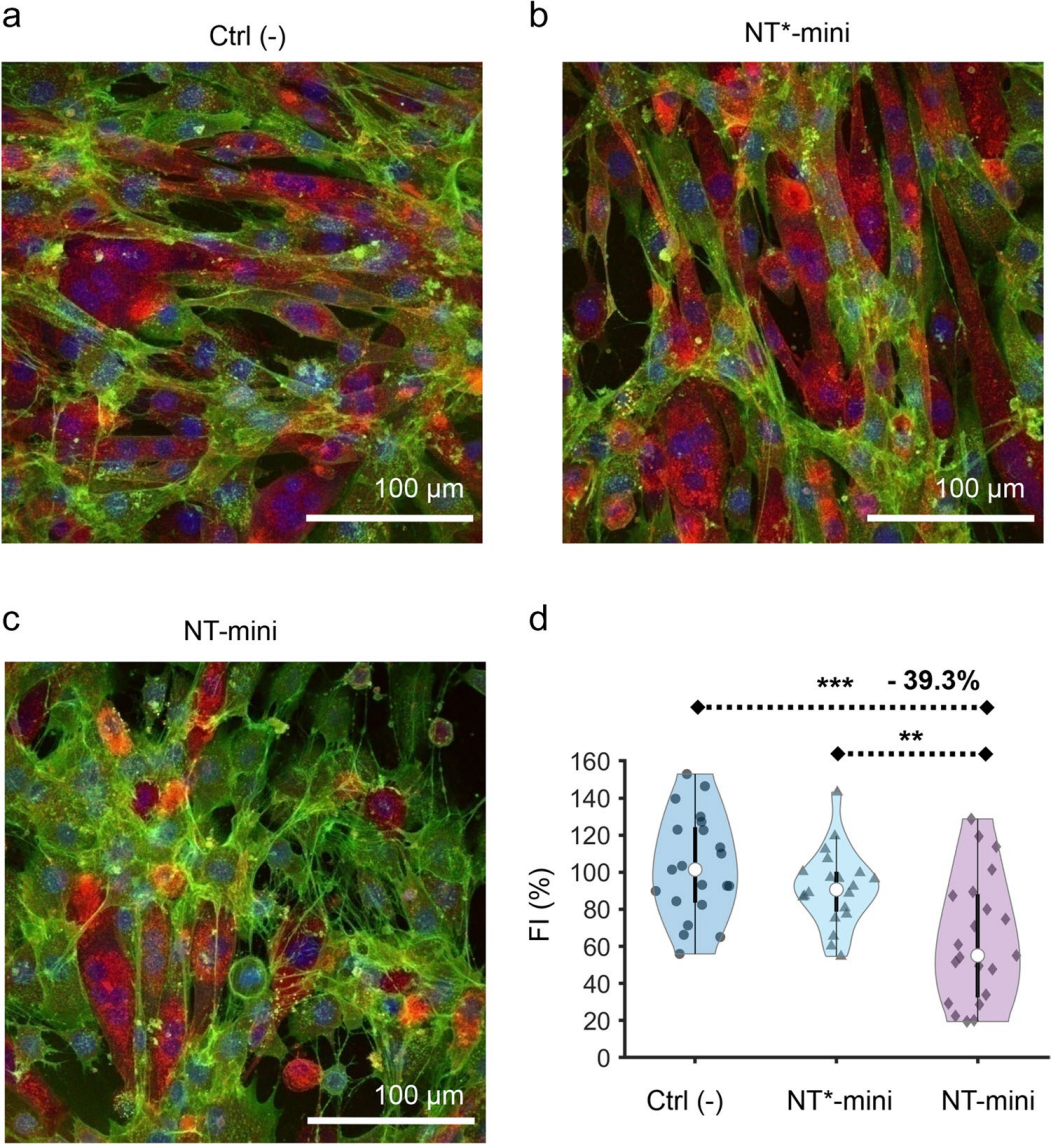
Effects of NT-mini on myotube formation. Representative images of C2C12 cultures differentiated in absence of NT-mini or NT*-mini (**a**), in presence of 50 ng/ml NT*-mini (**b**), or 50 ng/ml NT-mini (**c**). All cell images are maximum intensity Z-stack projections generated with ImageJ. Comparative violin plot of fusion indices normalized to non-treated controls (a, Ctrl (-)) (**d**). Treatment with NT-mini hampers myotube formation causing an approximate 39.3% reduction in myoblast fusion, as compared to non-treated controls and NT*-mini treated cultures. White dots indicate sample median, boxes show interquartile range, and whiskers indicate 1.5*interquartile range. Individual data points are shown in black circles (NT), triangles (NT*-mini), and diamonds (NT-mini). Statistical significance was determined by comparing mean values using a one-way ANOVA coupled with a Bonferroni multiple comparison test. All statistical analyses were performed with Prism 6.01 (GraphPad): **p ≤ 0.01; ***p ≤ 0.001. Violin plot was generated with matlab using the “Violinplot-Matlab” extension. Data were collected and analyzed from 21 images per condition obtained from independent triplicate (*n* = 3) experiments

Treatments with NT-mini had no visible impact on AChR distribution, which appeared generally diffuse throughout myotube membranes. Small AChR clusters were observed from time to time but seemed to have no relation with NT-mini treatments and were also found in presence of NT*-mini or in non-treated controls. However, when myotube formation was assessed based on the relative FIs, NT-mini was seen to inhibit the process. Whereas the inactive NT*-mini showed no significant variation compared to non-treated controls, NT-mini’s activity was associated with a significant (≈39.3%, *p* < 0.001) drop in FI (Fig. 5), indicating a reduction in myotube formation. This may offer an explanation for the muscle fiber loss reported in NT-overexpressing mice [11] and hints at a more direct involvement of NT in the process than might have been previously supposed.

### NT-Mini Regulates Hippocampal Neuron Functioning

**Fig. 6:**
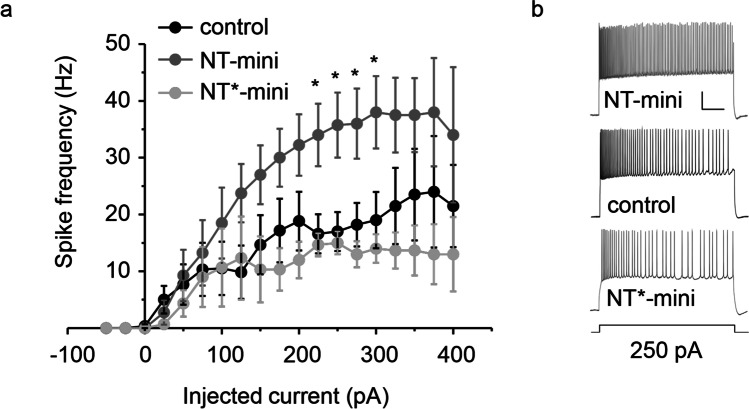
Effects of NT-mini on neuronal excitability. **a** Input/output relationship of hippocampal neurons with voltage responses elicited from Vrest using step current injection. The plot shows the relationship between average spike frequency (calculated over 1 s) and the injected current intensity for neurons recorded in the control condition (black), treated with NT-mini (dark grey), and NT*-mini (light grey). The average frequencies reached by NT-mini-treated neurons are significantly higher than controls and NT*-mini-treated neurons (*p < 0.05, for both distributions). Data is presented as the mean of replicate experiments (control *n* = 4, NT-mini *n* = 6, NT*-mini *n* = 3) and error bars indicate the standard error of the mean (MSE). **b** Examples of voltage responses elicited by 250 pA step current injection for a neuron treated with NT-mini (above), a control neuron (middle), and a neuron treated with NT*-mini (bottom). Scale bar: 20 mV/500 ms

Considering the stimulatory effect of NT on hippocampal neurons [10], we decided to address the impact of NT-mini (and NT*-mini) on hippocampal CA1 neuronal excitability. Experiments were performed by treating hippocampal slices with exogenously supplied recombinant proteins. Hippocampal CA1 pyramidal neurons showed enhanced intrinsic excitability after pre-incubation with NT-mini (Fig. 6), compared to controls (at 250 pA current injection step: 35.7 ± 5.7 Hz *n* = 4 vs. 17.0 ± 3.4 Hz *n* = 6, respectively, *p* = 0.021). The average resting membrane potential (Vrest: − 53.8 ± 2.4 mV in controls and − 55.4 ± 4.3 mV in NT-mini treated slices, *p* = 0.746) and spike threshold (− 46.2 ± 1.8 mV in controls and − 48.5 ± 2.6 mV in NT-mini treated slices, *p* = 0.480) were not significantly different. Conversely, the pre-incubation and treatment with NT*-mini did not significantly affect neuronal intrinsic excitability (15.0 ± 2.0 Hz, *n* = 3, *p* = 0.691), Vrest (− 60.0 ± 4.2 mV, *p* = 0.218), or spike threshold (− 41.2 ± 0.6 mV, *p* = 0.120), compared to control.

## Discussion

Proteases are known to play essential roles in processes of synapse formation and rearrangement, both in the CNS and at peripheral synapses like NMJs [5]. In this regard, the NT-agrin enzyme–substrate pair represents a shared signaling pathway associated with synaptic rearrangement of central and peripheral synapses. Agrin has long been known to play a direct role in synaptic organization [10, 53–59]; however, the contributions of NT to the same processes (agrin-dependent or otherwise) are generally less understood. While a number of observations have tentatively associated NT activity with synaptic plasticity [10–12, 18, 60], a more detailed molecular understanding of NT functionality and its interplay with agrin is lacking. This limitation has been compounded by the historically difficult recombinant production of full-length NT [16]. In this context, the comparatively simple production in HEK293 cells of our engineered NT-mini construct (spanning NT’s SP and most proximal SRCR domain) offered the opportunity to extensively investigate the molecular implications of NT’s activity and its regulatory mechanisms.

Despite its truncated nature, NT-mini conserved catalytic activity, and was capable of processing the β-cleavage site when presented on both agrin-like substrates and synthetic peptides (mM affinity) of different lengths, albeit with much lower efficiency. Shortening the assayed peptide-substrates (6-mer to single Lys) evidenced how NT-mini retains the specificity characteristic of the full-length NT, with the shorter peptides (4-mer and 3-mer) yielding the slowest reaction velocities, and the single Lys substrate remaining unprocessed. The longer 6-mer and 5-mer substrates showed preferential cleavage, with the latter seemingly highlighting a catalytic “sweet-spot” representing a plausible minimal catalytic recognition sequence (-GLVEK-). However, the “complete” substrate recognition mechanism of NT likely relies on multiple long-range contacts on portions of the substrate distant from the cleavage sequence. This is likely reflected in the increased 6-mer cleavage rates in the presence of heparin, and in the significantly faster processing observed with the agrin-like substrates.

Surprisingly, NT-mini’s response to the α-site mimicking peptide was significantly different, as we were unable to detect any significant degree of processing. This stands in stark contrast with our observations with the glycosylated recombinant AGR-124 substrate which, instead, showed that NT-mini is capable of α-cleavage. The difference in activity between AGR-124 and the synthetic α-peptide could be explained as the result of NT’s high specificity, providing further evidence that cleavage of agrin by NT is strongly dependent on additional exosite interactions extending far beyond the recognized cleavage sequences. This seems to be of particular importance for the α-site, hinting at a more stringent control of NT activity at that location, and one which might involve the complex glycosylations located along the various domains constituting the agrin C-terminus. Furthermore, we cannot exclude that such a multi-point recognition system might involve not only agrin domains and/or specific post-translational modifications (such as heparan/chondroitin sulfates), but also the NT accessory domains not included in NT-mini.

Regardless of the low affinities observed, NT-mini’s processing of short synthetic peptides allowed us to model and interrogate NT activity in a range of conditions, mapping specific high-affinity binding sites for Ca^2+^ and heparin within its two C-terminal domains. This is especially true regarding heparin, as it was possible to evidence picomolar affinity binding only in the SP domain.

Alongside these observations, our previously reported calcium-binding properties of the murine NT SRCR3 domain [24] seem to indicate that calcium binding by NT is likely to occur at multiple sites throughout the protein. Our combined results on Ca^2+^dependent modulation of enzymatic activity and thermal stabilization of NT-mini unambiguously indicate the presence of a high-affinity Ca^2+^ binding site(s) within the SP domain complemented by those likely to be found in the preceding SRCR domains [24, 61–63]. This is supported by the significantly different Ca^2+^ concentrations we have found to be effective in stabilizing or activating NT-mini. The latter provides a direct readout of Ca^2+^-SP domain interactions, and the former represents a measure of multiple Ca^2+^ binding events. Furthermore, our investigation of the NT binding specificity for Ca^2+^ yielded novel insights into the response of this enzyme to another bivalent metal ion essential in the CNS. When assaying the effects of zinc, NT-mini showed enhanced resistance to thermal denaturation (more so than with calcium), but a dramatic decrease in its catalytic activity. This might hint at a possible dual regulation of NT-mini (and therefore NT) mediated by the local synaptic concentrations of different bivalent ions. Calcium would serve as an enhancer of catalytic activity, while zinc might be responsible for its downregulation. Given the significant importance of synaptic Ca^2+^ and Zn^2+^ within the CNS [64–66], we speculate that NT might be capable of responding in vivo to different stimuli. This could, in turn, allow for spatio-temporal fine-tuning of NT’s activity not only based on neuronal activity, but also in relation to the fluctuations in zinc/calcium currents. Something which would be very much in agreement with the documented importance of NT activity for CNS/PNS synaptic plasticity [10, 12, 14, 18].

However, calcium, zinc, and heparin are not the only factors that likely modulate NT activity. Indeed, assaying NT-mini’s catalytic competence on engineered C-terminal fragments of agrin revealed a substrate-modulated activity dependent on the agrin splicing variations present on the substrate. This could be a further modulatory node, possibly partially independent from NT itself but strongly tied to the relative abundance of agrin splice variants within specific cellular contexts. Different cleavage rates could result in the accumulation of different agrin fragments, with potentially different biological roles, in a manner directly dependent on the local availability, distribution, and presence of substrate variants. In turn, that would lead to interesting regulatory possibilities that might account for agrin’s multiple reported biological roles as a consequence of subtle modulation of agrin-mediated signaling [10, 12, 57, 67–79].

Given the highly cooperative modulation of NT enzymatic activity, its effect on agrin signaling, and its involvement in processes of synaptic reorganization, it is surprising that deregulation of the NT-agrin axis has been linked to a relatively small number of pathological states [11, 12, 60, 80]. One of the few known pathogenic implications of NT deregulation is centered on the formation and maintenance of NMJs. Overexpression, and thus hyperactivity, of NT in mice has been documented to cause an acceleration in the NMJ life cycle, leading to premature synapse degradation correlated with generalized muscular weakness and wasting [11]. However, it is difficult to say if this phenotype originates primarily from the presynaptic compartment as a consequence of excessive NT activity. It might, instead, be subordinated to a reduction in muscle contraction caused by impaired NMJ signaling. Our experiments performed with NT-mini on C2C12 myotubes offer new insights into this process. Specifically, our data indicate a direct impact of NT-mini’s enzymatic activity on myotube formation, something which may explain the muscle wasting observed in NT-overexpressing mice [11].

A separate consideration can be made regarding NT deregulation in the CNS. There, the reduction of enzymatic activity is seemingly associated with subtle alterations in synaptic homeostasis leading to mental retardation [12] and/or neurodegeneration [60]. Our investigations on hippocampal CA1 neurons showed that NT activity can be responsible for the direct modulation of neuronal excitability. Therefore, in a physiological context, the multi-factorial regulation of NT might play an immediate role in fine-tuning signal processing, and alterations in its functionality could upset the delicate balance of CNS excitability leading to loss of synaptic plasticity or even excitotoxicity.

## Conclusion

The characterization of the precise biological roles and significance of the NT-agrin axis has suffered severe limitations due to the lack of reliable reagents for in vitro and cellular assays. Our work on the generation and characterization of NT-mini and the associated library of agrin fragments expands upon the molecular understanding of this nervous system serine protease. Our results shed light on previously unknown features, indicating that efficient processing of agrin by NT requires recognition of a three-dimensional substrate through long-range contacts, and that such activity is differentially regulated by different metal ions (Ca^2+^/Zn^2+^). These observations hint at the possibility of delicate fine-tuning along a NT-agrin signaling axis, potentially correlating to a wide spectrum of cellular responses. Such hypothesis not only is consistent with the impacts of both NT and agrin on CNS and PNS synaptic plasticity [10, 12, 14, 18, 57, 67, 69], but also offers plausible insight into the wider expanse of agrin’s biological functions [70–79, 81]. Surprisingly, our data also indicate that NT’s accessory domains do not significantly contribute to this regulatory aspect of the signaling pathway, as explicit modulation of proteolytic activity appears traceable to the SP domain alone. The non-catalytic domains of NT might play a role in restraining/directing NT activity within the synapse [82], a feature likely essential to preserve the physiological balance of NT-agrin interplay which, when upset, can have far-reaching consequences directly affecting muscle formation and neuronal excitability. Together with previously published data, our results highlight how NT is likely more than a highly specificity serine protease. Rather, it might represent a “hub” capable of directing agrin-induced biological responses via fine-tuned multi-factorial modulation of its own proteolytic activity.

## Supplementary Information

Below is the link to the electronic supplementary material.

Supplementary file1 (PDF 3092 KB)

## Data Availability

All datasets generated and analyzed for this work are available from the corresponding authors upon reasonable request.
