# Supplementary material for: Deconstruction of Neurotrypsin Reveals a Multi-factorially Regulated Activity Affecting Myotube Formation and Neuronal Excitability
Source: Mol Neurobiol. 2022 Oct 5;59(12):7466–85. doi: 10.1007/s12035-022-03056-2 (PMC9616769; doi:10.1007/s12035-022-03056-2)
Supplement: Supplementary file 1 — Supplementary file1 (PDF 3092 KB) [file 12035_2022_3056_MOESM1_ESM.pdf]

Supplementary information

Deconstruction of Neurotrypsin reveals a multi-factorially regulated activity affecting myotube formation and neuronal excitability.

Anselmo Canciani[1,*], Cristina Capitanio[1,#], Serena Stanga[2,§], Silvia Faravelli[1], Luigi Scietti[1,XX], Lisa Mapelli[3], Teresa Soda[3], Egidio D'Angelo [3,4], Pascal Kienlen-Campard[2], Federico Forneris[1,*]

[1] The Armenise-Harvard Laboratory of Structural Biology, Department of Biology and Biotechnology, University of Pavia, Via Ferrata 9/A, 27100 Pavia (Italy).
http://fornerislab.unipv.it
[2] Aging and Dementia Research Group, CEMO Department, Institute of Neuroscience, UCLouvain, B-1200 Brussels (Belgium)
[3] Department of Brain and Behavioral Sciences, University of Pavia, Via Forlanini 6, 27100 Pavia (Italy).
https://dangelo.unipv.it/
[4] IRCCS Mondino Foundation, Via Mondino 2, Pavia (Italy)

[#] *Present address: Molecular Machines and Signaling, Max Planck Institute of Biochemistry, 82152 Martinsried, Germany.*
[§] *Present address: Neuroscience Institute Cavalieri Ottolenghi, 10043 Orbassano (TO), Italy; Department of Neuroscience Rita Levi Montalcini, University of Turin, 10126 Turin, Italy.*
[XX] *Present address: Biochemistry and Structural Biology Unit, Department of Experimental Oncology, IRCCS European Institute of Oncology (IEO), Via Adamello 16, 20139 Milan (Italy).*

** To whom correspondence should be addressed: Federico Forneris, e-mail:* federico.forneris@unipv.it; *Anselmo Canciani, e-mail:* anselmo.canciani@iusspavia.it

Supplementary Table 1: List of primers used for construct amplification. Restrictions sites are underlined. Modified codon for Ser-825-Ala mutation indicated in italics.

Construct	Primer sequence (5'-3')
**NT-mini**	**Fwd** AAAGGATCCGGTTTTCCTGTCAGACTGATGGATGG
	**Rev** TAAGCGGCCGCCAGTTTGGTGACACTTTTTATCCAAGGTACAAAGG
**NT*-mini**	**Fwd** *GCT*GGAGGACCACTCATGTGTGA
	**Rev** GTCTCCCTGGCAGCTGTCCA
**Agrin LG2-LG3**	**Fwd** AAAAGGATCCCCCTTCCTGGCTGACTTCAAC
	**Rev** AAAAGCGGCCGCTGGGGTGGGGCAGGGCCG
**Agrin y4**	**Fwd** CGTGTGTTGGGGGAGTCCCCGAAAAGCCGCAAAGTTCCGCACACCGTCCTCAACCTG
	**Rev** CAGGTTGAGGACGGTGTGCGGAACTTTGCGGCTTTTCGGGGACTCCCCCAACACACG
**Agrin z8**	**Fwd** GCCAACGAAATTCCGGTGGAGAAGGCACTGCAGAGCAA
	**Rev** CCGGAATTTCGTTGGCCAGTTCGCTCTCGGTCACAGCGTTG
**Agrin z11**	**Fwd** CTGGATAGCGGCGCGCTGCATAGCGAGAAGGCACTGCAGAGCAA
	**Rev** CGCGCCGCTATCCAGGGTTTCCGGGCTCTCGGTCACAGCGTTG
**Agrin z19**	**Fwd** TGGCCAACGAAATTCCGGTGCCGGAAACCCTGGATAGC
	**Rev** GCTATCCAGGGTTTCCGGCACCGGAATTTCGTTGGCCA

Supplementary Table 2: synthetic substrates used for NT-mini characterization.

	Peptide α	Peptide β	β (5 mer)	β (4 mer)	β (3 mer)	Lys-pNa
**Sequence**	GPPVER-pNa	KGLVEK-pNa	GLVEK-pNa	LVEK-pNa	VEK-pNa	K-pNa
**Source**			*China Peptides*			*BACHEM*

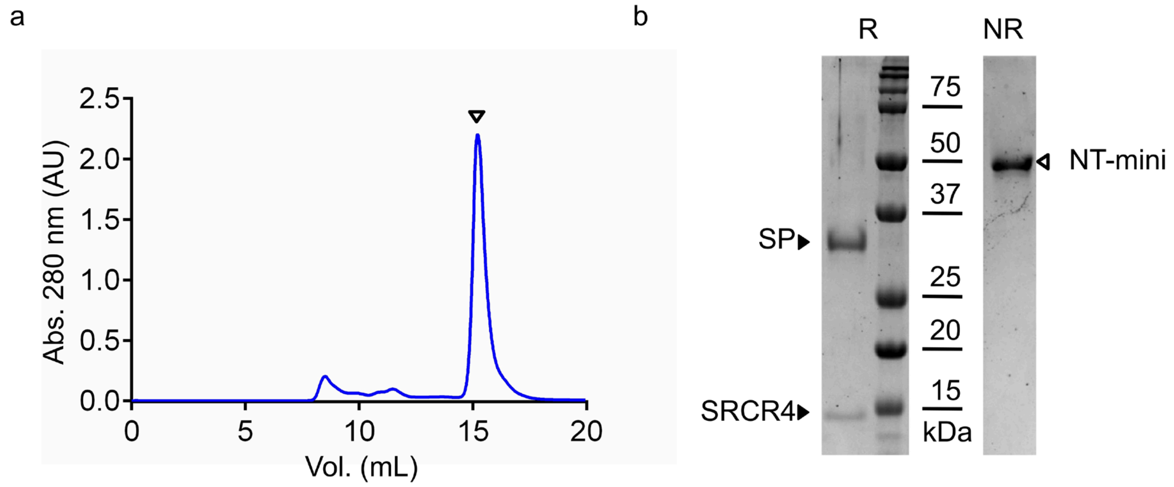

Supplementary figure 1 - Summary of NT-mini purification.

(a) Size exclusion chromatography (SEC) representative of the final purification step for NT-mini. The protein is visible as a single sharp peak (white triangle) well separated from higher MW contaminants. (b) SDS-PAGE gel representative of the final purified product. The two-chain active form of NT-mini (white arrow) is visible as a single band close to the 50 kDa MW marker in non reducing conditions (NR). In reducing (R) conditions the disulfide bond bridging the SP and SRCR4 domains is broken, and these are visible (black arrows) below the 37 kDa and 15 kDa markers, respectively.

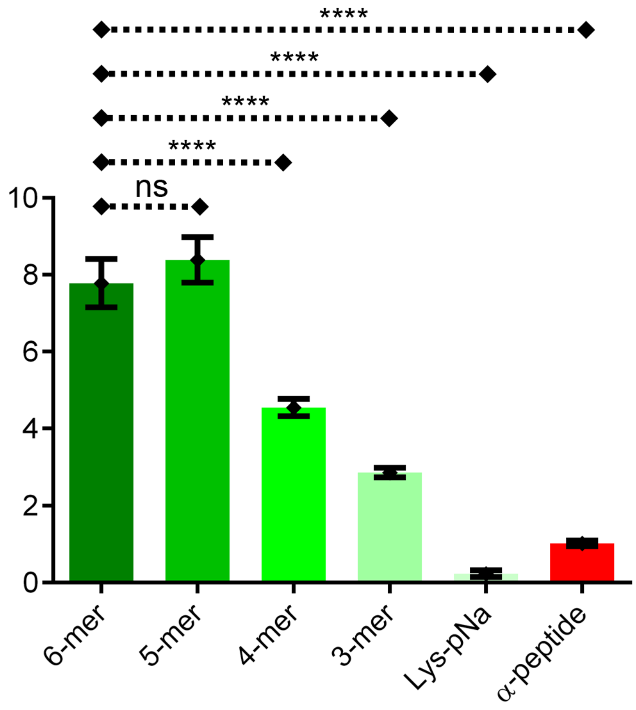

Supplementary figure 2 - Comparison of NT-mini activity on synthetic peptides

Comparative V_0 plot of shortened (6-mer to 3-mer) β peptide substrates, and alternative synthetic substrates: peptide α (-GPPVER-) or single Lys pNa conjugates. Substrate concentration was fixed at 1 mM for initial investigations. Plot was generated in graphpad (Prism 6.01); data is shown as the mean of triplicate ($n = 3$) independent experiments, error bars represent the standard deviation (SD) of each data point. ****, $p \leq 0.0001$; ns, non-significant.

a

b

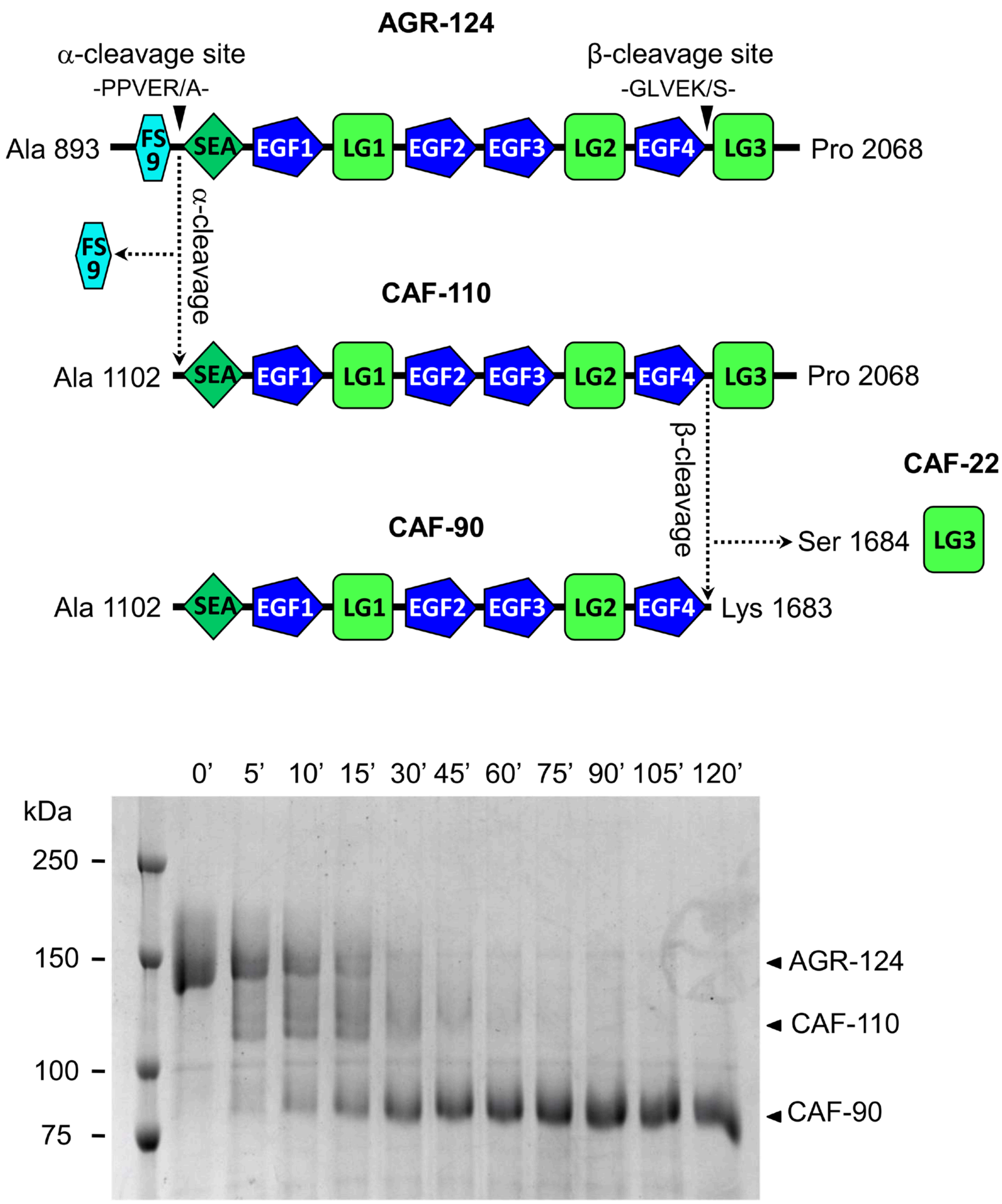

Supplementary figure 3 – Evidence for alpha cleavage.
(a) Schematic representation of the AGR-124 α-substrate boundaries, domain organization, and α/β cleavage C-terminal agrin fragment (CAF) products of 110 (CAF-110), 90 (CAF-90) and 22 (CAF-22) kDa. FS: Follistatin-like, SEA: sperm, enterokinase and agrin, EGF: Epidermal growth factor-like, LG: Laminin globular. (b) SDS-PAGE showing a time-resolved digestion of AGRIN-124 by NT-mini. The substrate (AGR-124) is processed to generate the intermediate (CAF-110) and final (CAF-90, CAF-22) cleavage products. All residue numbering refers to human agrin (O00468-1).

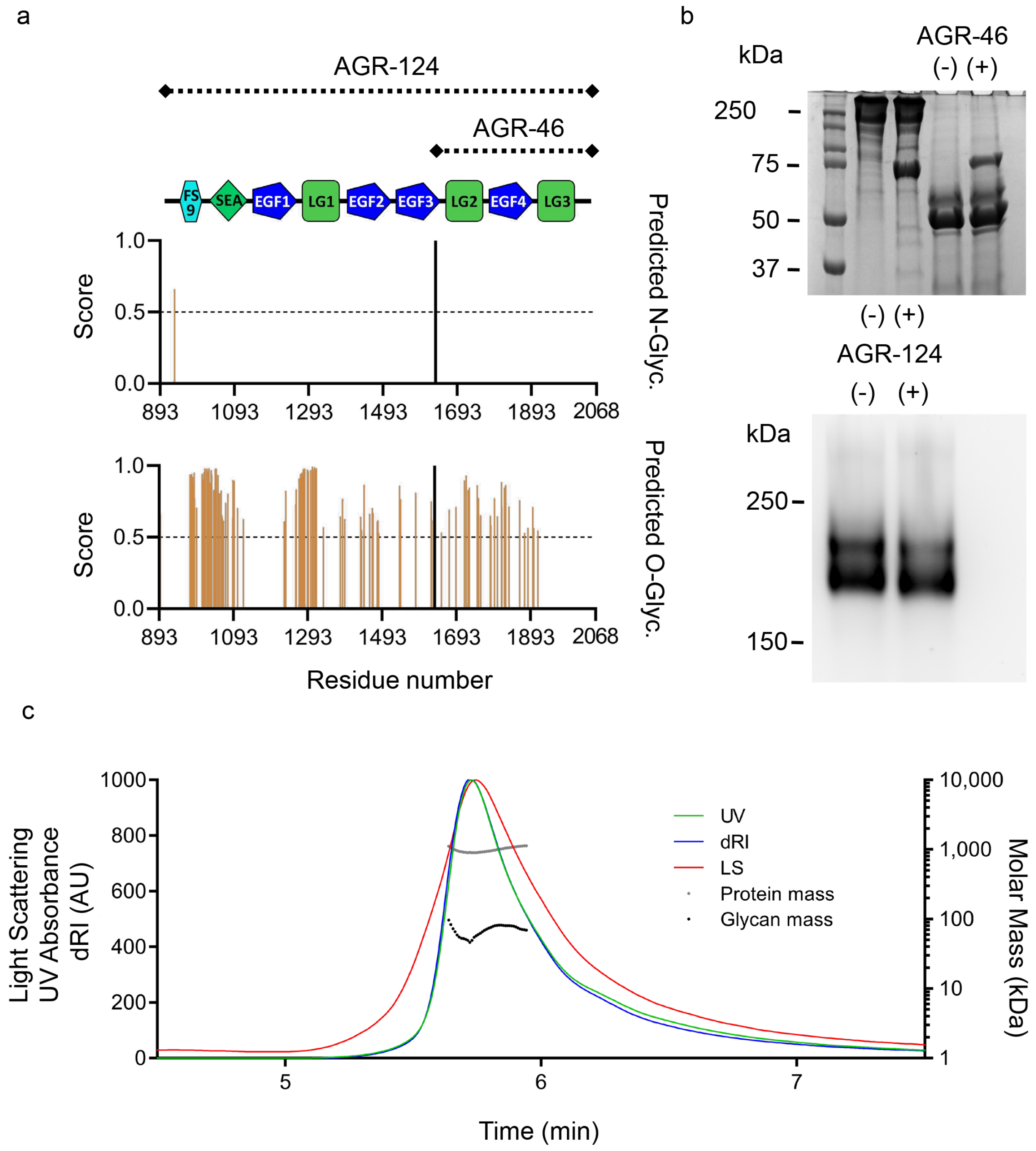

Supplementary figure 4 – Analysis of glycosylations of recombinant agrin fragments.
(a) *in silico* prediction of N-linked (top panel) and O-linked (bottom panel) glycosylations of the human AGR-124 construct and its shorter AGR-46 fragment. Reported histogram bars (orange) refer to predicted glycosylations by the NetNGlyc and NetOGlyc servers with confidence higher that 50%. (b) SDS-PAGE analysis of PNGAse-F digestion of recombinant AGR-46 and AGR-124 constructs, highlighting only minimum shifts for the AGR-124 construct upon glycosydase treatment visible only when using a low-percentage SDS-PAGE (bottom panel). (c) SEC-MALS analysis of recombinant AGR-124. The chromatogram shows traces corresponding the UV absorbance at 280 nm (green), the differential refractive index (blue) and the light scattering intensity (red) around the main elution peak. The computed molar mass for the protein fraction (grey) and glycans (black) are overlayed to the chromatogram, consistently showing an overall 10% contribution of the glycans to the total molar mass of the eluting protein.

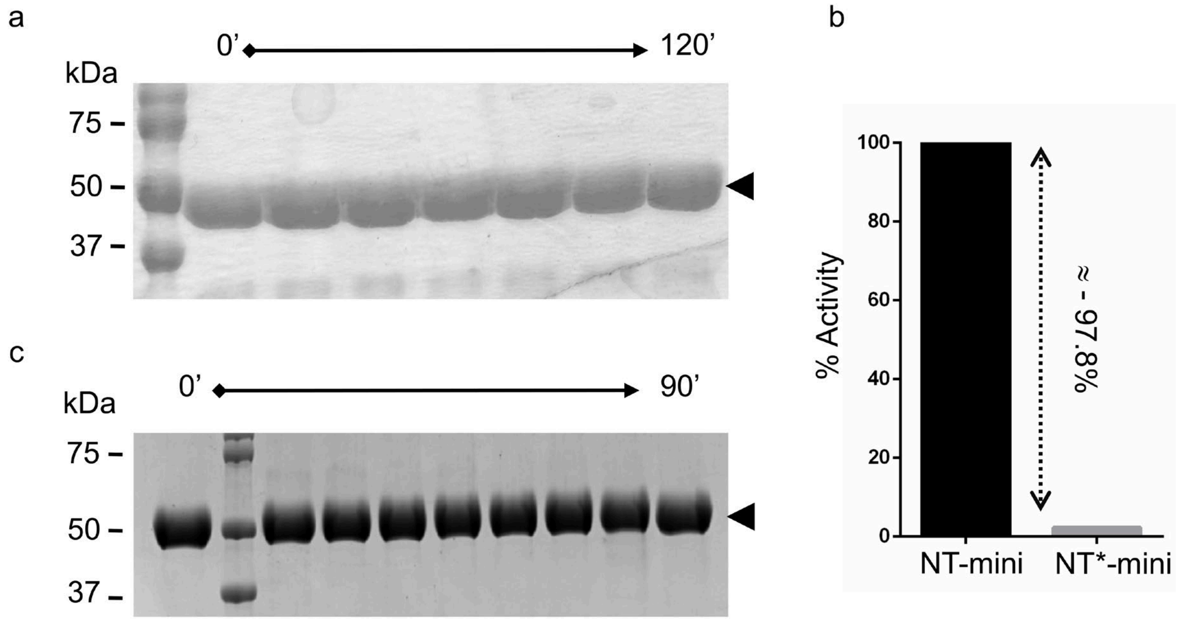

Supplementary figure 5 – Inactivation of NT-mini by Zn^{2+} and assessment of inactivity of NT*-mini.

(a) SDS-PAGE showing a time-course of non-digestion of an agrin-like substrate (black arrow) by NT*-mini. (b) Activity of NT*-mini on the reference synthetic 6-mer β-peptide as compared to NT-mini. % activity is normalized to the V_0 of NT-mini. (c) SDS-PAGE of a time a resolved digestion assay of an AGR-46 substrate (black arrow) by NT-mini in presence of 0.2 mM $ZnCl_2$. The presence of zinc strongly inhibits NT-mini leaving the substrate unprocessed.

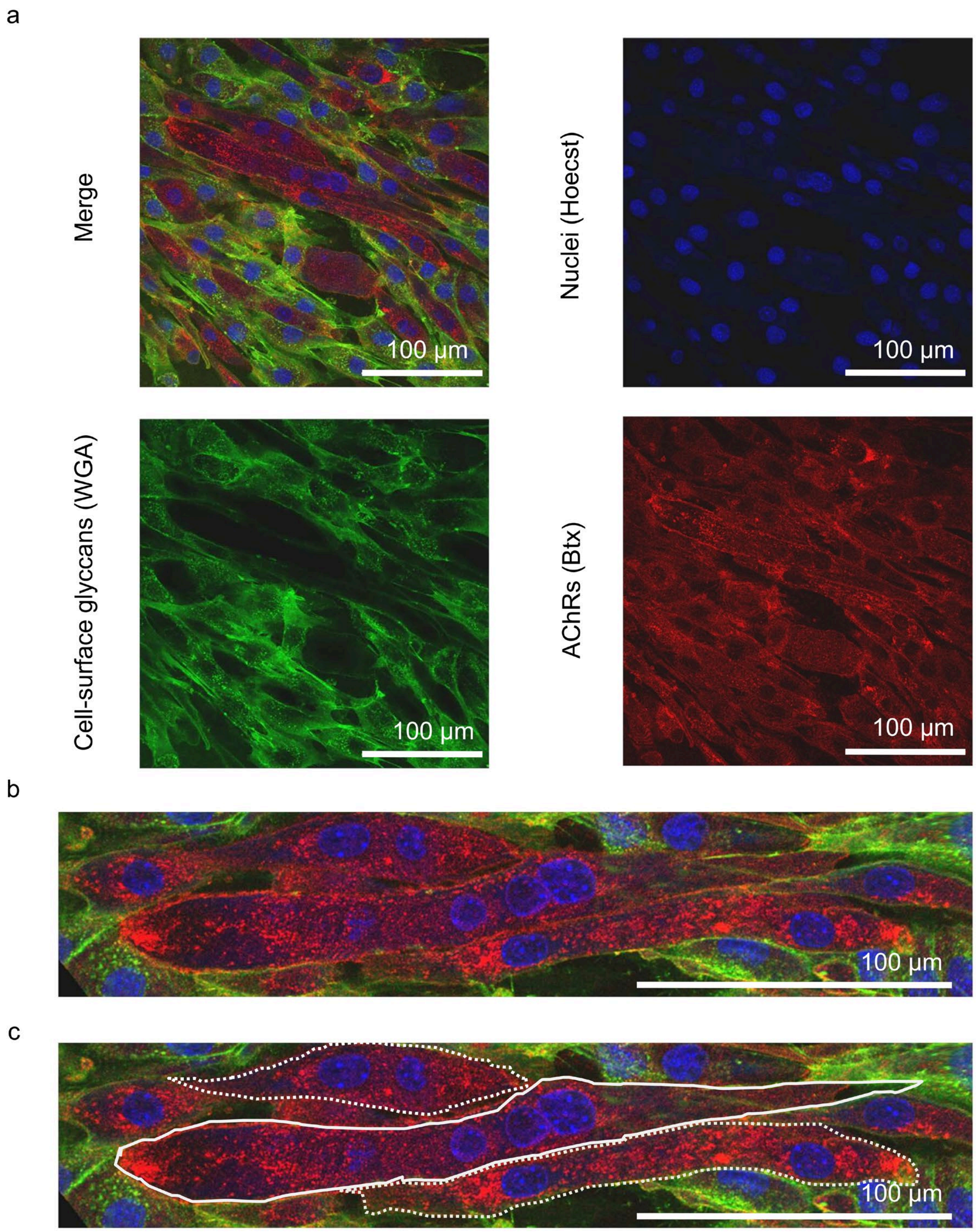

Supplementary figure 6 - C2C12 staining and myotube identification.
(a) Single confocal Z-stack slice images, collected at 40x magnification, representative of myotube/myoblast staining. Nuclei are stained with hoecst (blue), cell-surface glycans are stained with a WGA-Alexa647 (wheat germ agglutinin-Alexa647) conjugate (green), and AChRs are stained with a Btx-Alexa594 (bungarotoxin-Alexa594) conjugate (red). (b) Section of the single Z-stack slice showing a mixed population of myoblasts and myotubes. (c) Myotubes (white outline) are identified as AChR positive cells containing 3 or more nuclei, while “myoblasts” (dotted white outline) contain 2 or less. Fusion indices are calculated as the fraction of total nuclei contained in myotubes.
